# Coat protein of partitiviruses isolated from mycorrhizal fungi functions as an RNA silencing suppressor in plants and fungi

**DOI:** 10.1038/s41598-022-11403-5

**Published:** 2022-05-12

**Authors:** Hanako Shimura, Hangil Kim, Akihiko Matsuzawa, Seishi Akino, Chikara Masuta

**Affiliations:** Research Faculty of Agriculture, Hokkaido University, Kita-ku, Kita 9, Nishi 9, Sapporo, 060-8589 Japan

**Keywords:** Virus-host interactions, Viral immune evasion

## Abstract

Orchid seeds depend on colonization by orchid mycorrhizal (OM) fungi for their germination; therefore, the orchids and OM fungi have long maintained a close relationship (e.g., formation of the hyphal mass structure, peloton) during their evolution. In the present study, we isolated new partitiviruses from OM fungi; partitivirus were separately found in different subcultures from the same fungi. Partitiviruses have been believed to lack an RNA silencing suppressor (RSS), which is generally associated with viral pathogenicity, because most partitiviruses isolated so far are latent in both plants and fungi. However, we found that the coat protein (CP) of our partitiviruses indeed had RSS activity, which differed among the virus isolates from OM fungi; one CP showed RSS activity in both plants and fungi, while another CP showed no activity. The family *Partitiviridae* include viruses isolated from plants and fungi, and it has been suggested that these viruses may occasionally be transmitted between plant and fungal hosts. Given that there are several reports showing that viruses can adapt to nonhost using strong RSS, we here discussed the idea that partitiviruses may be better able to migrate between the orchid and fungus probably through the pelotons formed in the orchid cells, if host RNA silencing is suppressed by partitivirus RSS.

## Introduction

The family Orchidaceae contains about 30,000 species and is the most diversified and largest plant family in monocotyledons^1–3^. One of their unique characteristics is a mycoheterotrophic nutritional requirement for seed germination; in current knowledge, all orchids rely on colonization by orchid mycorrhizal fungi (OM fungi) for germination^4,5^. The seeds of orchid are very small and consist only of a seed coat and an undeveloped embryo without any storage tissues such as endosperm and cotyledons found in seeds of other plants. When OM fungi infect orchid seeds in their natural state, mycelial masses called the “peloton” are formed in the intracellular spaces of orchid cells, then the seeds germinate using nutrients obtained from degradation of the peloton^6,7^. Most OM fungi are classified as basidiomycetes, which include the form-genus *Rhizoctonia* (sensu lato) and ectomycorrhizal fungi in woody plants^8^. Although some orchids are partners of certain fungal species, many orchids are not specific for any mycorrhizal fungus, and little is known about the specificity of the orchid–fungus interactions and the factors that influence the establishment of symbiotic relationships^9,10^.

Mycoviruses are viruses that infect fungi; double-stranded RNAs (dsRNAs) of either their genomes or replicative intermediates are isolated from infected fungi^11,12^. Mycoviruses have a variety of effects on host fungi including reduced growth, reduced spore formation, and altered virulence of pathogenic fungi^13^. The effects of mycoviruses on the characteristics of OM fungi are not well understood, and we wondered whether the mycoviruses of OM fungi can affect the symbiotic relationship between the fungi and orchids. In our preliminary experiments using the OM fungi isolated from a wild *Cypripedium* (lady’s slipper orchid) in Hokkaido, Japan (*Cypripedium macranthos* var. *rebunense*)^14^, we found that several mycoviruses actually infected the OM fungi that are highly effective at inducing germination of *C*. *macranthos* var. *rebunense*. There is no evidence that mycoviruses derived from mycorrhizal fungi play any roles in the process of symbiosis establishment. However, there are some examples of the mycoviruses in OM fungi-related phytopathogenic fungi (*Rhizoctonia solani*) affecting the interactions between plants and fungi. For example, *R. solani* infected with a partitivirus showed a reduction in mycelial growth and hypovirulence to the host plant rice^15^. The nature of OM fungi infected with mycoviruses may be also affected by viral infection, and accordingly the symbiotic interactions between the OM fungi and host orchid may be changed.

In metagenomic analyses to detect viruses in OM fungi using RNA-seq, Ong and their group detected several partitiviruses^16^. The viruses in the family *Partitiviridae* have been detected in plants, fungi and protozoa. In the previous classification in *Partitiviridae* according to the ICTV ninth report^17^, viruses that infect only fungi are classified into the genus *Partitivirus*, while viruses that infect plants are classified into the genera *Alphacryptovirus* and *Betacryptovirus* and called as “cryptic virus”. As the sequence information accumulated, we learned that “partitiviruses in fungi” and “cryptic viruses in plants” are phylogenetically closely related, and they are not clearly distinguished based on their host organisms. All these viruses are now called partitiviruses and have been classified in five genera: *Alphapartitivirus*, *Betapartitivirus*, *Gammapartitivirus*, *Deltapartitivirus* and *Cryspovirus*^18,19^. Recently, two new genera, *Epsilonpartitivirus* and *Zetapartitivirus*, have been proposed^20,21^, and yet another new genus might be included in the family *Partitiviridae*^22^. Partitiviruses comprise one of the largest families including many virus species. Partitiviruses contain two linear dsRNA genomes ranging from 1.4 to 2.4 kb^19^. In fungi, these viruses are transmitted intracellularly during cell division, hyphal anastomosis and sporogenesis, whereas plant partitiviruses are transmitted intracellularly via seeds through infection to ovule or pollen^18,19^. Most partitiviruses have been found to infect their host organisms without causing any detectable effects^19^. However, several partitivirus species have been reported to cause hypovirulence in some phytopathogenic fungi^20,23–26^.

At least some mycoviruses, like other viruses, can overcome the host’s defense response for infection and propagation in the host’s cells by suppressing RNA silencing; a conserved gene silencing process in eukaryotes is considered to be a defense mechanism in fungi against mycoviruses^27^. For example, Hammond et al. analyzed three *Aspergillus* mycoviruses and found that two virus strains (1816 and 341) can suppress RNA silencing in the *Aspergillus* cells^28^. Cryphonectria parasitica hypovirus 1 (CHV1) encodes a papain-like protease p29, which has some sequence similarity to the helper component proteinase (HC-Pro) of potyvirus^29^. p29 is the first-identified RNA silencing suppressor (RSS) in a mycovirus and functions as RSS by inhibiting the expression of key factors in the host RNA silencing pathway including Dicer and Argonaute proteins^30,31^. S10 of Rosellinia necatrix mycoreovirus 3 (RnMyRV3) is another example of mycovirus RSS, which showed the RSS activities in fungal cells as well as in plant cells^32^. In addition, the ORF2 protein of Fusarium graminearum virus 1 (FgV1) has RSS activity, which suppresses the expression of the Dicer2 and AGO1 genes^33^. These results together suggest that quite a number of mycoviruses seem to have a strategy to overcome RNA silencing in host fungi for their successful infection, whereas no partitivirus with RSS has been identified so far.

A mycovirus was detected in an endophytic fungus that can induce thermal tolerance in the host plants, and the elimination of the virus from the fungus resulted in a loss of the fungal thermal tolerance-inducing ability^34^. Although the detailed mechanism for the thermal tolerance was not elucidated, this study demonstrated that mycoviruses could control the mutualistic interactions between the fungus and host plant under certain circumstances. In this way, it is conceivable that viruses in OM fungi may affect the specific relationship between plants and fungi considering that orchids and OM fungi have evolved in their close relationship. We here focused on partitiviruses in OM fungi isolated from *Cypripedium*. Because the genera *Alphapartitivirus* and *Betapartitivirus* include viruses isolated from both plants and fungi, it has been considered that the viruses may occasionally be transmitted between these two organisms in different kingdoms. The objectives of this study are (1) to ascertain whether partitiviruses have RSSs, and if so, (2) to determine whether it functions as a strong RSS in different hosts (plants and fungi). In this study, we analyzed the RSS activity in OM fungi partitiviruses to overcome antiviral RNA silencing and discussed the relationship between viral RSS activity and host adaptation.

## Results

### Partitiviruses are detected in the mycorrhizal fungi of *Cypripedium*

**Figure 1:**
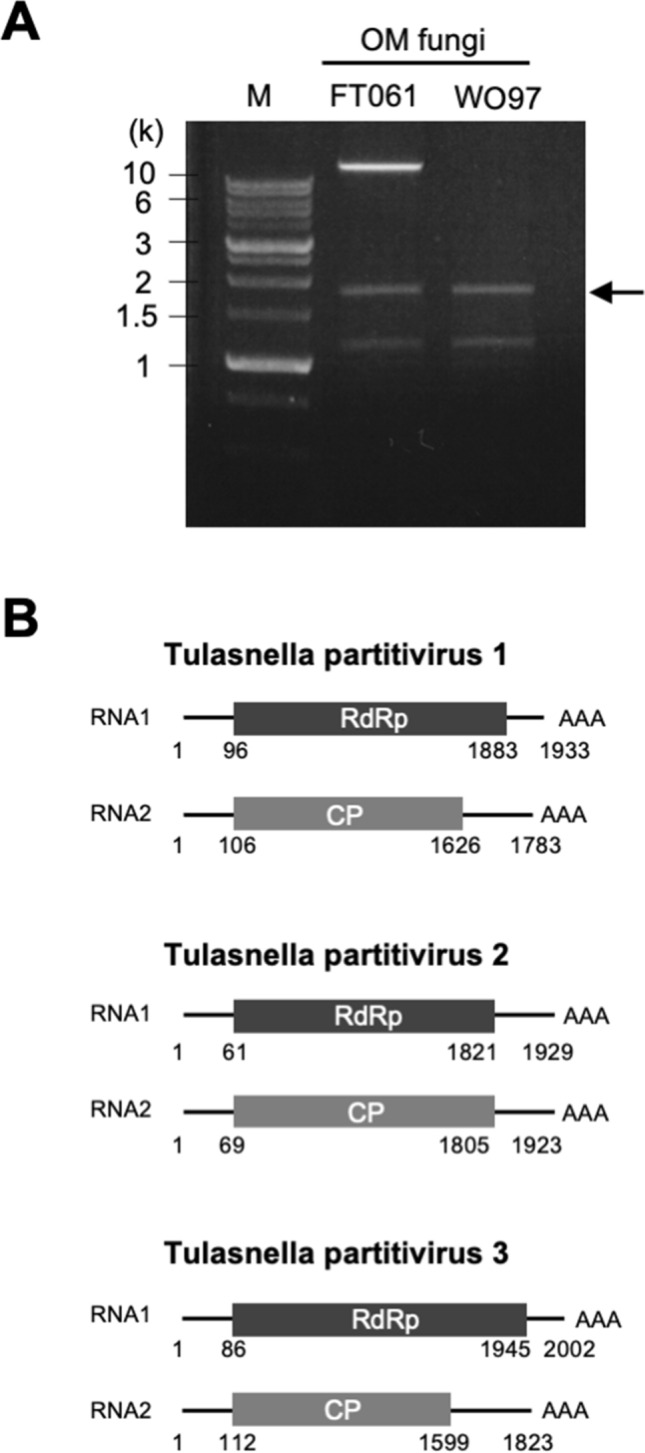
Partitiviruses isolated from OM fungi of *Cypripedium macranthos* var. *rebunense*. (**A**) dsRNAs extracted from two OM fungal strains (WO97 and FT061), which were detected in agarose gel electrophoresis. (**B**) Genome organization of Tulasnella partitivirus 1–3. The longer RNA (RNA 1) has a single open reading frame (ORF) encoding an RdRp and the shorter RNA (RNA 2) has a single ORF encoding a coat protein (CP).

dsRNAs were extracted from two strains of OM fungi (WO97 and FT061) and detected as a clear band(s) around 2 kb in an agarose gel (Fig. 1A, Supplementary Fig. S1). A band of dsRNA larger than 10 kb was also detected from strain FT061 (Fig. 1A, lane FT061). We focused on the bands at ~ 2 kb, cut out them from the gel, cloned them, and determined the sequences; the 5' and 3' end sequences were determined using Rapid Amplification of cDNA Ends (RACE). Viral sequences were also confirmed based on contig sequences obtained from RNA-seq data using the cDNAs from the dsRNAs in two OM fungi. Our sequencing results revealed that the ~ 2 kb dsRNAs were partitivirus-derived, and RNA1 encoding the RNA-dependent RNA polymerase (RdRp) and RNA2 encoding the coat protein (CP) were paired based on the homology between the two molecules in the 5' and 3' end sequences (Supplementary Fig. S2). In OM fungi used in this study, three kinds of partitivirus sequences were obtained; Tulasnella partitivirus 1 and Tulasnella partitivirus 2 were detected from strain WO97, and Tulasnella partitivirus 2 and Tulasnella partitivirus 3 were detected from strain FT061 (Fig. 1B). These results indicate that the OM fungi used in the experiment were infected with multiple partitiviruses. On the other hand, from the RNA-seq results, we also found that the number of partitiviruses differed even among the same fungal strains; e.g., one derivative isolate of strain WO97 had only Tulasnella partitivirus 1, whereas one to eight partitiviruses were detected among derivative isolates of strain FT061; we believe that the initial search in FT061 using cloning-based virus detection was not exhaustive. Mycoviruses may have been heterogeneously distributed in the hyphae samples originated even from the same fungus in the process of subculture. Because the hyphae grown in liquid culture for the dsRNA isolation were derived from a small portion of a fungal colony on the plate, this procedure may have caused variation in viral detection.

### Partitiviruses from the mycorrhizal fungi of *Cypripedium* belong to the genus *Alphapartitivirus*

**Figure 2:**
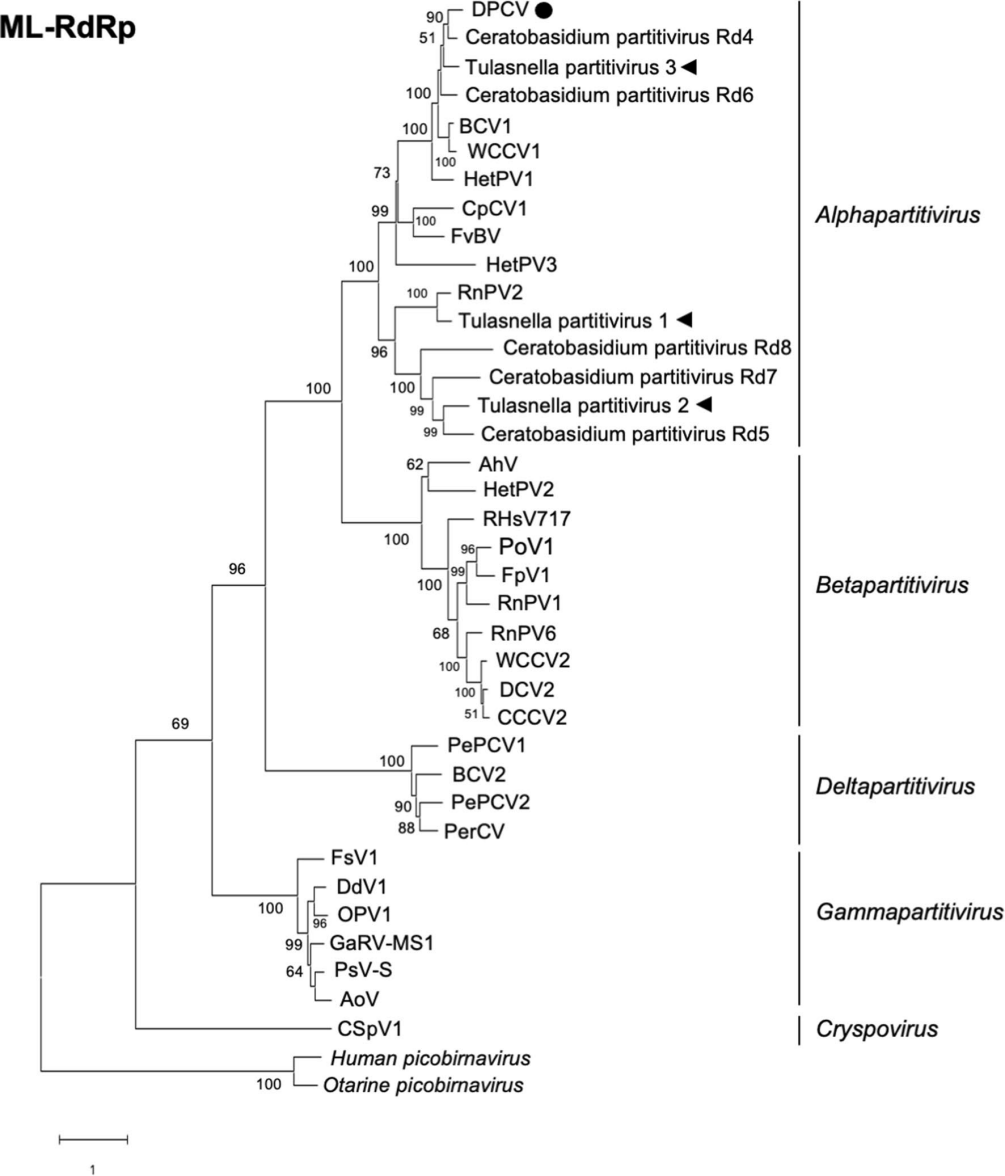
Phylogenetic tree based on the amino acid sequences of RdRp of partitiviruses and constructed by the ML method. Bootstrap values > 50 are shown at the branch points. The evolutionary-distance scale is the number of substitutions per site. Tulasnella partitiviruses detected in this study are indicated by black triangles. The partitivirus detected in an Australian wild orchid (DPCV) is shown with a black circle.

**Figure 3:**
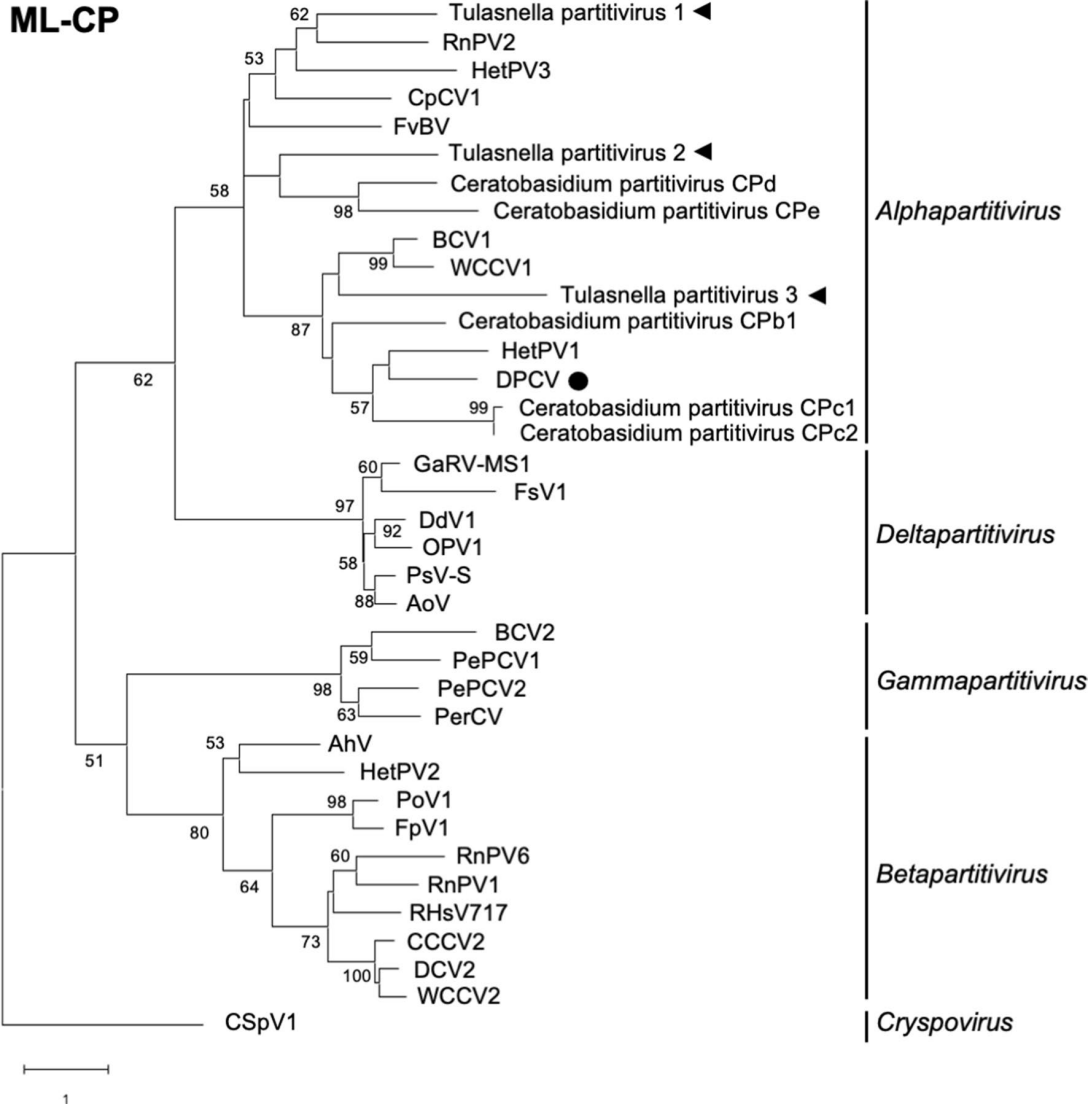
Phylogenetic tree based on the amino acid sequences of the CP of partitiviruses and constructed by the ML method. Bootstrap values > 50 are shown at the branch points. The evolutionary-distance scale is the number of substitutions per site. Tulasnella partitiviruses detected in this study are indicated by black triangles. The partitivirus detected in an Australian wild orchid (DPCV) is shown with a black circle.

A BLAST search using amino acid sequence of RdRp showed that Tulasnella partitivirus 1 had a high similarity to alphapartitivirus (e.g., 74% identity to RdRp of Gyromitra esculenta partitivirus 1, and 70% identity to RdRp of Heterobasidion partitivirus 20). RdRp of Tulasnella partitivirus 2 showed 58% identity to that of Erysiphe necator partitivirus 2, and 57% to Sarcosphaera coronaria partitivirus. For RdRp of Tulasnella partitivirus 3, Gaeumannomyces tritici partitivirus 2 (67%) and Rhizoctonia oryzae-sativae partitivirus 2 (66%) were listed as top-hit species. Our phylogenetic analyses of the Tulasnella partitivirus 1 to 3 sequences using the ML method and phylogenetic tree construction confirmed that all these viruses belong to the alphapartitivirus group. In the phylogenetic tree of RdRp (Fig. 2), Tulasnella partitivirus 1 and Tulasnella partitivirus 2 were separated from a group that included Tulasnella partitivirus 3, but each Tulasnella partitivirus clustered independently. In the phylogenetic tree of CP (Fig. 3), the viruses in the alphapartitivirus group were roughly similar in the topology of RdRp tree, but not separated as clearly as the RdRp. Among the few reports of mycoviruses detected in OM fungi^16,35–37^, one report showed alphapartitiviruses and betapartitiviruses infection in OM fungi belonging to *Ceratobasidium* spp. isolated from an Australian orchid (*Pterostylis sanguinea*)^16^. In addition, Diuris pedunculata cryptic virus (DPCV) was isolated from Australian endemic orchid, *Diuris pedunculata*^38^. In the phylogenetic tree of RdRp, DPCV and some alphapartitiviruses from Australian OM fungi were clustered with Tulasnella partitivirus 3, and Tulasnella partitivirus 2 was clustered with other alphapartitiviruses from Australian OM fungi.

### Partitivirus CP in the OM fungi of *Cypripedium* had RSS activity in plant tissues

**Figure 4:**
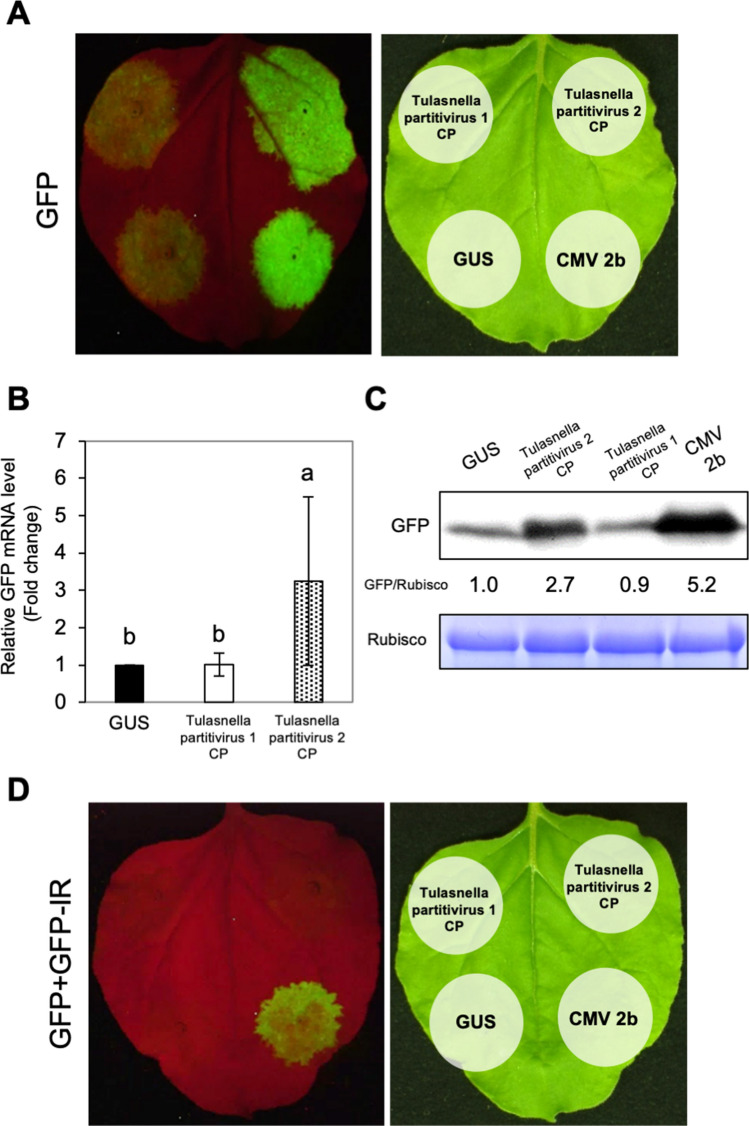
RSS activity of partitivirus CPs derived from OM fungi. (**A**) Assessment of RSS activity of the partitivirus CPs against sense RNA-mediated GFP silencing. Tulasnella partitivirus 1 CP or Tulasnella partitivirus 2 CP was expressed with GFP in *N. benthamiana* leaves by agroinfiltration. RSS activity of partitivirus CPs was assessed by comparing GFP intensities under UV light at 5 days post agroinfiltration (dpa). (**B**) Real-time RT-PCR for comparison of relative GFP transcript levels. The leaf samples from agroinfiltrated patches were collected at 5 dpa. Data were compared as values of fold-change relative to the control (GUS), and the values were analyzed on log-transformed data by Tukey’s multiple test (*P < 0.05). Means and confidence intervals are shown in the graph. Different letters above the bars indicate a significant difference among the experimental groups. (**C**) Western blot analysis to compare GFP levels using anti GFP antibodies. The Rubisco large subunit (Rubisco) is shown as a loading control for each sample. (**D**) Assessment of RSS activity of the partitivirus CPs against dsRNA-mediated GFP silencing. Each partitivirus CP was expressed with GFP and GFP-IR in *N. benthamiana* leaves by agroinfiltration. The GFP intensities were observed under UV at 2 dpa.

**Figure 5:**
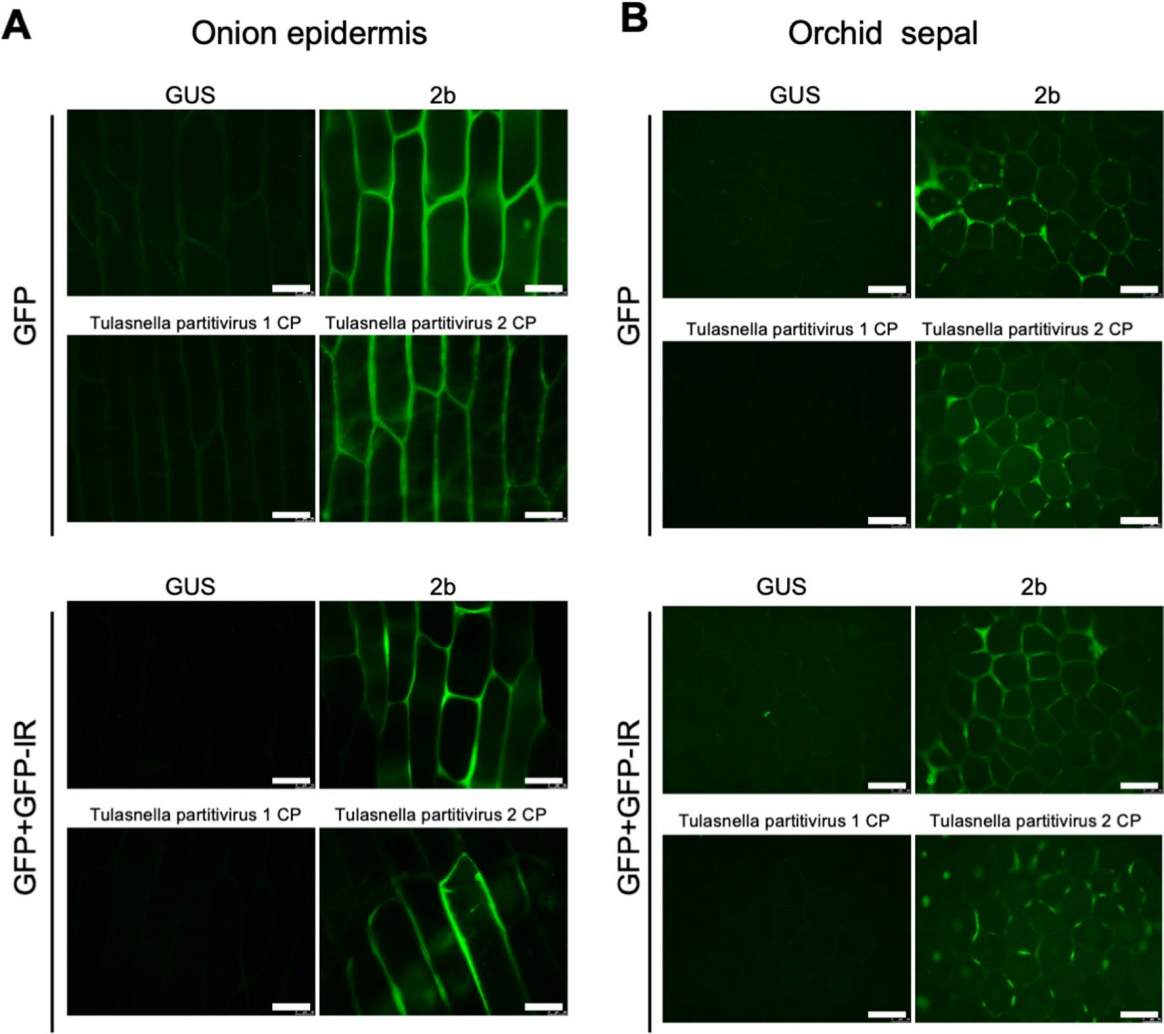
RSS activity of partitivirus CPs in onion and orchid cells. GFP and partitivirus CPs were co-expressed with or without GFP-IR by agroinfiltration in onion epidermis (**A**) and *Phalaenopsis aphrodite* sepal tissues (**B**). Images were taken under UV using an epifluorescence microscope at 3 dpa. Scale bars: 50 μm. CMV 2b and GUS were used as a positive and negative control, respectively.

**Figure 6:**
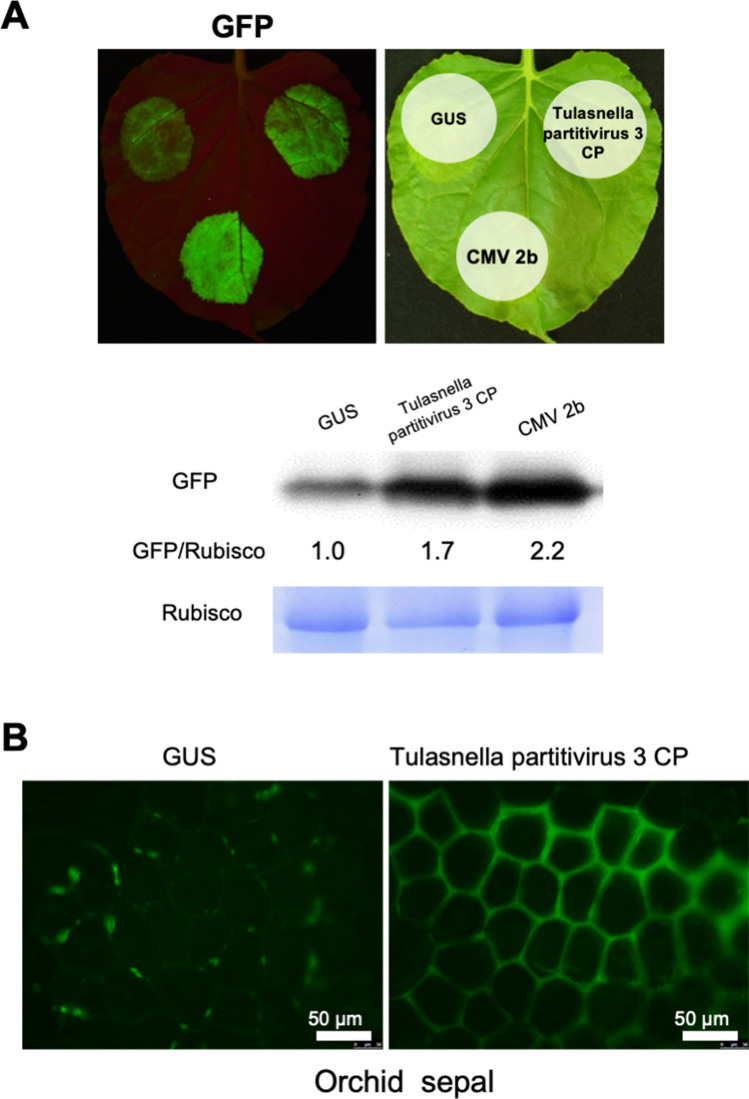
RSS activity of Tulasnella paritivirus 3 CP. Tulasnella paritivirus 3 CP was co-expressed with GFP in *N. benthamiana* leaves (**A**) or *Phalaenopsis* sepal tissues (**B**) by agroinfiltration. GFP fluorescence was assessed at 5 dpa for *N. benthamiana* or at 3 dpa for *Phalaenopsis*. For agroinfiltration in *N. benthamiana*, CMV 2b and GUS were used as a positive and negative control, respectively, and the level of GFP was compared by western blot analysis. For agroinfiltration in *Phalaenopsis*, GFP fluorescence was observed using an epifluorescence microscope. Scale bars: 50 μm.

Partitiviruses are dsRNA viruses and have been thought not to be a target of RNA silencing in host cells because viral replication occurs within stable particles (T1 = 1 capsid)^39^. Although partitiviruses have been suggested to be able to counteract host RNA silencing^40^, partitivirus proteins have not been proven to show RSS activities. We hypothesized that any protein product(s) of a partitivirus would have RSS activity, and that CP, which is always exposed outside of the particle, would be more likely than RdRp, which is inside the particle, to serve as an RSS. In line with this hypothesis, we first tested the CP for RSS activity using agroinfiltration, a transient assay system using *Nicotiana benthamiana* leaves. When RNA silencing was induced by the overexpression of the GFP sense RNA, Tulasnella partitivirus 1 CP was found to have no RSS activity, while Tulasnella partitivirus 2 CP showed strong RSS activity at a level comparable to cucumber mosaic virus (CMV) 2b used as a positive control (Fig. 4A). Elevated levels of GFP mRNA and protein were confirmed by qRT-PCR and western blots, respectively (Fig. 4B,C, Supplementary Fig. S3). However, when silencing was induced by dsRNA synthesized from the inverted-repeat construct of GFP, none of the partitivirus CPs had RSS activity (Fig. 4D). Next, we used an agroinfiltration assay system using the epidermis of onion, which is in the same order Asparagales as orchids, because the viral RSS activity seemed to be host-dependent according to our previous study^41^. The results showed that Tulasnella partitivirus 1 CP did not have RSS activity, but Tulasnella partitivirus 2 CP had RSS activity in the onion tissues not only for sense RNA but also for dsRNA (Fig. 5A). We also tested whether the RSS activity could be detected in orchid cells after agroinfiltration of sepal tissues of *Phalaenopsis aphrodite*. As expected, the results were similar to those for onion (Fig. 5B). We also confirmed that co-agroinfiltration with GUS did not negatively interfere with the GFP expression in the tissues of *N. benthamiana*, onion and orchid (Supplementary Fig. S4). Using the assay system with *N*. *benthamiana* and *P*. *aphrodite*, we further examined RSS activity of the CP of Tulasnella partitivirus 3 and found that Tulasnella partitivirus 3 CP also had RSS activity in the two plant species (Fig. 6, Supplementary Fig. S5).

### Partitivirus CP in the OM fungi has RSS activity in fungi

**Figure 7:**
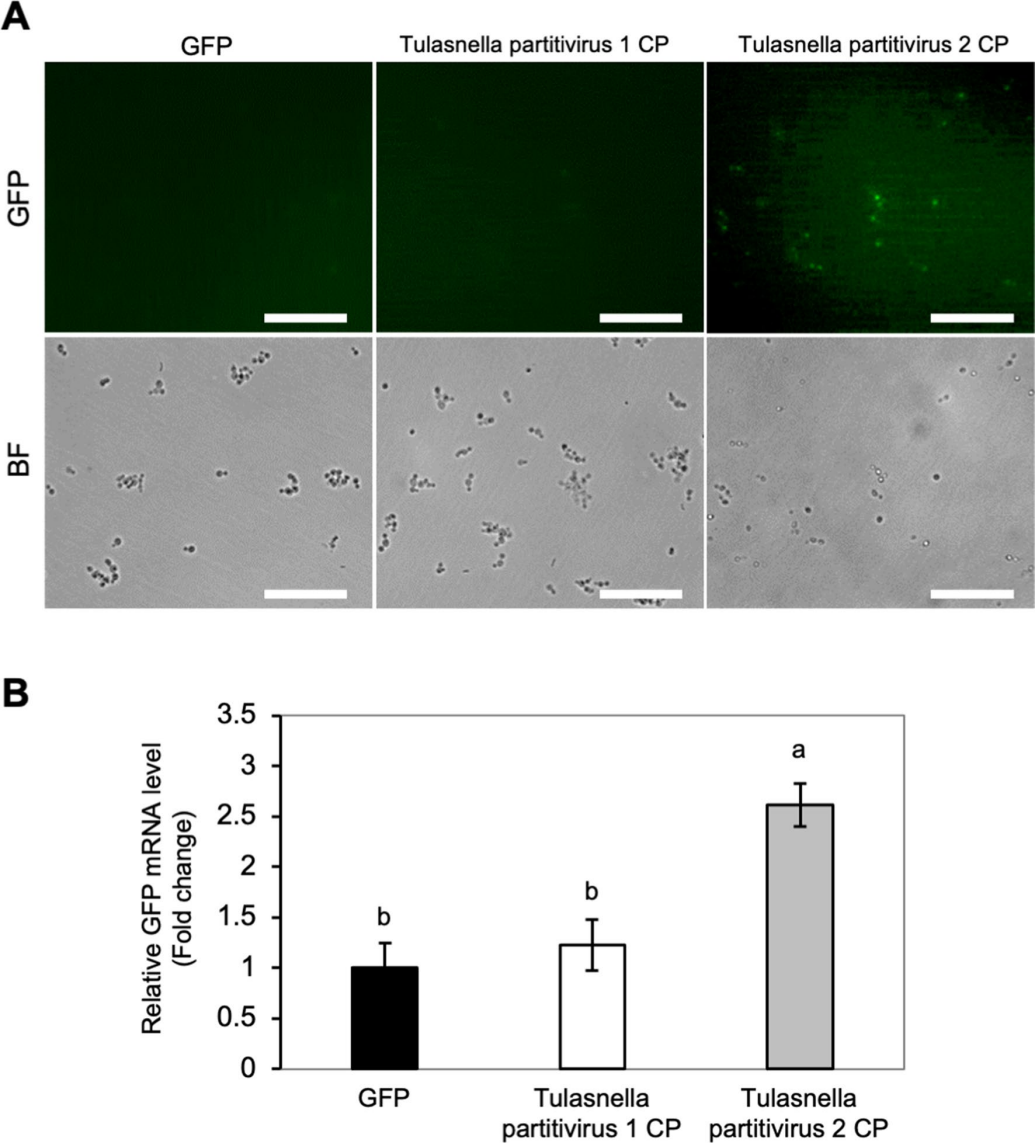
RSS activity of partitivirus CPs in *Rhizoctonia* protoplasts. (**A**) Micrographs of *Rhizoctonia* protoplasts expressing GFP and partitivirus CPs. Protoplasts of *R. solani* AG4-HG2 were co-transformed with pMF280-EGFP, CMV RNA4A harboring partitivirus CP and GFP dsRNA. Images were taken at 60 h after transfection using an epifluorescence microscope. Scale bars: 50 μm. (**B**) Relative GFP transcript accumulation levels in *Rhizoctonia* protoplasts. The GFP mRNA levels in the protoplasts were analyzed by real-time RT-PCR after 60 h. Ribosomal RNA was used as an internal control. Data were compared as values of fold-change relative to the control (GFP), and the values were analyzed on log-transformed data by Tukey’s multiple comparison test (*P* < 0.05). Means and confidence intervals were shown in the graph. Different letters above each bar indicate significant differences.

We then tested whether the RSS activity observed in Tulasnella partitivirus 2 could be detected not only in plants but also in fungi by using protoplasts of *Rhizoctonia solani*, a related species of the OM fungi. First, we constructed a transfection system for *Rhizoctonia* protoplasts based on the RSS activity assay system using plant protoplasts^42^. As a result, although the fungal protoplast cells were very small and difficult to observe even under the microscope, we could identify clear RSS activity of the CP of Tulasnella partitivirus 2 as shown in Fig. 7; elevated GFP fluorescence was observed in Tulasnella partitivirus 2 CP-transfected protoplasts (Fig. 7A), and GFP mRNA levels were also high in Tulasnella partitivirus 2 CP-expressing cells (Fig. 7B). In contrast, Tulasnella partitivirus 1 CP had no RSS activity in fungal protoplasts. For Tulasnella partitivirus 1 CP without RSS activity, considering the possibility that the intact CP was not synthesized in the transfected cells, we transfected protoplasts with the CP-GFP fusion gene to observe the GFP fluorescence. As a result, we observed GFP fluorescence and confirmed that Tulasnella partitivirus 1 CP was indeed synthesized in the fungal cells (Supplementary Fig. S6). Based on the number of cells with GFP fluorescence, we calculated the transfection efficiency to be about 42.6%.

### RSS activity of partitivirus CP varies among isolates

To get an idea of whether the RSS activity of the partitivirus CP varies with the isolate derived from the fungus or plant, we also analyzed the RSS activity of CP of DPCV that was an alphapartitivirus detected in an Australian wild orchid^38^. We found that DPCV CP did not show RSS activity against both sense- and dsRNA-induced silencing in agroinfiltrated *N*. *benthamiana* plants (Supplementary Fig. S7A), but it did in onion and orchid cells (Supplementary Fig. S7B,C). In the fungal protoplast assay, DPCV CP had no obvious RSS activity compared to Tulasnella partitivirus 2, although GFP mRNA levels were elevated in DPCV CP-transfected cells (Supplementary Fig. S8). These results together indicate that CPs of the partitiviruses have differential RSS activities ranging from none to strong and lacked host-specificity regardless of whether the virus was isolated from a fungus or a plant.

## Discussion

In this study, we identified alphapartitiviruses derived from orchid mycorrhizal (OM) fungi, which had ability to induce seed germination of *Cypripedium*^14^. Among a few studies of mycoviruses in OM fungi, virus infection in orchids and OM fungi has been well studied in Australia^16,35,36^. Interestingly, phylogenetic analyses of partitivirus showed that partitiviruses derived from OM fungi that are endemic to the northernmost island of Japan and those from Australian OM fungi were included in the same clade. Unlike the other partitiviruses, only partitivirus in OM fungi may have been subjected to a specific selection pressure, which must have been generated in the symbiotic interaction, so that they evolved similarly into the same group. As more information become available on viruses infecting OM fungi isolated from various regions, we will better understand the role of viruses in the symbiosis between orchids and fungi.

We here also showed that CP of the partitiviruses derived from the OM fungi has RSS activity; Tulasnella partitivirus 2 CP showed RSS activity not only in plant cells (tobacco, onion and orchid cells) but also in fungal cells. The specialized peloton in the intracellular spaces of cortical tissues in the embryo or in developed roots of orchids is surrounded by an extension of the plasma membrane of orchid cell. At the cellular interface between the orchid and fungi, orchids are thought to control hyphal growth by producing antibiotic substances like phytoalexins and to absorb nutrients derived from degradation of the peloton (Supplementary Fig. S9). In such a situation, mycelial degradation within the orchid tissues would allow free movement of micromolecules between orchid and fungus. Viral RNAs or particles are also quite likely to pass between the plant and fungus. For example, CMV could pass from infected plants to phytopathogenic fungi *Rhizoctonia solani*^43^. Furthermore, Bian et al. reported that when plants infected with a plant virus (CMV or tobacco mosaic virus) were infected with a fungus (*F. graminearum*) containing a mycovirus (CHV1), both viruses could easily move between the two hosts, suggesting the plant-fungal-mediated routes for host-switching of fungal and plant viruses in nature^44^. However, even if there is a situation where virus can move between the orchid and fungal cells, whether the virus can replicate in the destination cells is another matter.

To invade a new host cell, the virus must overcome the host resistance mechanism. In a study of this possibility, protoplasts of *N*. *benthamiana* were transfected with partitivirus particles isolated from *Penicillium aurantiogriseum*, but the virus was unable to replicate^45^. However, in *N*. *benthamiana* protoplasts expressing HC-Pro, a potyvirus RSS, there was an increase in the viral RNA, suggesting that the partitivirus replicated even in cells that are not the original host^45^. The implication of this result is that the existence of an RSS and its suppressive activity is important for partitiviruses to switch host organisms. Rosellinia necatrix partitivirus 2 (RnPV2) isolated from the plant pathogen *Rosellinia necatrix* has been shown to be targeted by host RNA silencing^46^. As for whether the partitivirus has an RSS, Rosellinia necatrix partitivirus 1 (RnPV1-W8) did not show RSS activity^32^. On the other hand, the presence of RSS activity was suggested recently for RnPV6 isolated from *R. necatrix*^40^. RnPV6 was found to be tolerant to host RNA silencing, because accumulation of genomic RNA of RnPV6 did not increase when the virus was introduced in RNA silencing-deficient mutant fungi^40^. In addition, TmPV1, a partitivirus isolated from the animal pathogen *Talaromyces marneffei*, inhibited players in the RNA silencing pathway in the infected fungal cells and also promoted the virulence of *T*. *marneffei* in mice^47^. Although many fungal partitiviruses and plant cryptic viruses have been reported so far, in most cases, the effect of the virus on the host has been considered to be rather small. Partitiviruses with strong RSS activity may emerge when the relationship between the fungus and its host is closely established such as in a pathogenic or symbiotic interaction. In our study, Tulasnella partitivirus 1 CP did not show RSS activity in any of the assay systems, while Tulasnella partitivirus 2 CP showed strong RSS activity in both plants and fungi. These results thus suggest that RSS activity of partitiviruses varies widely. In plant viruses, RSS activity has been shown to be the determinant of the persistence of viral infection and pathogenicity to the host^48^. Plant viral RSSs can also affect viral host adaptation. For example, pepper ringspot virus RSS enabled potato virus X to infect the nonhost plant *Arabidopsis*, suggesting that RNA silencing plays a role in restricting a nonhost-adapted virus^49^. In the same way, partitivirus with strong RSS activity may be able to dominate its host fungus, affecting its virulence to the host plant. When we isolated partitiviruses from OM fungi, strain WO97 was infected with Tulasnella partitivirus 1 and Tulasnella partitivirus 2, and strain FT061 had Tulasnella partitivirus 2 and Tulasnella partitivirus 3. Considering that CP of Tulasnella partitivirus 1 had no RSS activity among the tested CPs, mixed infection would be an important factor for virus survival. Even if a partitivirus has no or very weak RSS activity, it might be able to switch to a new host through a mixed infection with a partitivirus that has strong RSS activity. How RSS diversity of partitivirus affects their host fungi will be the next topic of our research interest.

The phylogenetic trees of RdRp and CP based on the amino acid sequences showed that the RdRp tree, in which branching was supported with high probabilities, was much more robust than the CP tree. This may be due to the low homology (~ 30% at the highest) among the partitivirus CPs. The low CP homology may be related to the wide host range of partitiviruses. According to the recent review by Petrzik^50^, reassortants are quite frequently generated in partitiviruses during mixed infections, so Tulasnella partitivirus 1 to 3 may not consist of specific pairs of RNA 1 and RNA 2 in mixed infections. Two studies using next-generation sequencing (NGS) technologies have revealed that partitivirus CP sequences are scattered throughout the host genome^51,52^, suggesting that the variability of the viral CP sequences and their differential RSS activities may be generated at least partly by recombinations between the viral CP and endogenous sequences. The virus will benefit from the genetic resources provided by recombination in the host cell, but the host cell might induce more easily RNA silencing against the invading partitivirus triggered by endogenous sequences. In any case, we believe that the acquisition of RSS during the viral evolutionary process would provide a great survival advantage for partitiviruses.

## Methods

### Plant materials

*Nicotiana benthamiana* plants, which have been maintained in Laboratory of Pathogen-Plant Interactions, Research Faculty of Agriculture, Hokkaido University, were grown at 25 °C with a 16-h day. Onion (*Allium cepa*) bulbs and potted plant of *Phalaenopsis aphrodite* were purchased at a market.

### Extraction, cloning and sequencing of dsRNAs from orchid mycorrhizal (OM) fungi

Two OM fungi (WO97 and FT061) have been isolated from roots and germinated protocorms of *Cypripedium macranthos* var. *rebunense*, respectively^14^ in Laboratory of Crop Physiology, Research Faculty of Agriculture, Hokkaido University, and now maintained in the same lab. From the ITS–5.8S sequences, these OM fungi are judged to be a species of the genus *Tulasnella* (Tulasnellaceae, Basidiomycota), but further identification of their species has not been done because their sporulation has not been confirmed. These fungi were propagated in oatmeal broth (2 g/L fine oatmeal powder without agar) for 10 weeks, and dsRNAs were extracted from fungi using a dsRNA extraction kit (ISOVIRUS, NIPPON GENE CO., LTD, Tokyo) and the manufacturer’s instruction. Alternatively, we extracted total RNA and then treated it with DNase and S1 nucleases to purify dsRNAs as described before^53^. For conventional cDNA cloning, first-strand cDNAs were synthesized using a random hexamer after denaturing dsRNAs by boiling, and dsDNAs were then synthesized using a PrimeScript double strand cDNA Synthesis Kit (Takara Bio, Shiga, Japan). The cDNAs were cloned into a T-vector (pGEM-T Easy vector, Promega) and Sanger-sequenced. At least three clones were sequenced to determine the full-length sequence. To determine the 5′ and 3′ end sequences, we used the 5′/3′ RACE kit, 2nd Generation (Roche Diagnostics, Mannheim, Germany). For RNA-seq, the extracted dsRNAs were sent to Hokkaido System Science (Sapporo, Japan) for RNA-seq analysis using a standard protocol. Briefly, the cDNA library was made from the dsRNAs using TruSeq RNA sample Prep Kit (Illumina). After purification and elimination of small molecules (< 200 bp) by AMPure XP beads, the library samples were applied to 100-bp paired-end sequencing using Illumina HiSeq 2000. Reads in each sample were subjected to adapter trimming and then used for de novo assembly using Trinity^54^ (http://trinityrnaseq.sourceforge.net/index.html, version 2013-2-25). After the raw sequence data were selected by filtering, only high-quality data were sorted by the index-tag sequence in the adaptor primer for each sample. Obtained contigs were annotated using Blastx+ (version 2.2.29+) against the amino acid databases of DDBJ viral (ftp://ftp.ddbj.nig.ac.jp/ddbj_database/dad/ddbjvrl1.DAD.fasta.gz). To confirm the viral sequences determined by RNA-seq, we performed RT-PCR to amplify the sequences expected from the information in the RNA-seq outcomes.

### Phylogenetic analysis

Phylogenetic trees of partitivirus RdRp or CP were created using MEGA X software^55^. The amino acid sequences were first aligned using MUSCLE, and the aligned sequences were used to construct the tree using maximum likelihood (ML) method under the rtREV + G + I + F model (for RdRp) or WAG + G + F model (for CP). The models with the lowest BIC and AICc values were automatically selected by the software, “Find best models (ML)” command in MEGA X, and used for the construction of the phylogenetic trees. Bootstrap values (> 50%) from 1000 replicates are shown on branches. The viruses and accessions used to construct the trees are listed in Supplementary Table S1.

### Analysis of RSS activity in plant species

For expression of partitivirus CPs in plant cells, CP genes were amplified by PCR with primer pairs PV1-CP5-Bam/PV1-CP3-Sc (for Tulasnella partitivirus 1), PV2-CP5-Bam/PV2-CP3-Sc (for Tulasnella partitivirus 2), or PV3-CP5-XbaI/PV3-CP3-Sc (for Tulasnella partitivirus 3), and then cloned into the pBE2113 binary vector using BamHI/SacI or XbaI/SacI restriction enzyme sites. The synthetic sequence of DPCV CP (GenBank: JX891460.1) was also cloned into pBE2113. Each GFP and GFP-inverted repeat (GFP-IR) construct was also cloned to pBE2113, respectively, to evaluate whether transient expression of the partitivirus CP suppresses RNA silencing against GFP. The plasmid constructs for the RSS activity assay using plant cells was shown in Supplementary Fig. S10. *Agrobacterium* strain KYRT1 was transformed with the recombinant plasmids, and the transformants were cultured in YEP broth (1% yeast extract, 1% bacto peptone and 0.5% NaCl). The harvested bacterial cells were suspended in MMA buffer (10 mM MgCl_2_, 10 mM MES and 0.2 mM acetosyringone). For inoculating *N*. *benthamiana* and *Phalaenopsis*, the concentration of bacterial suspension was adjusted to OD_600_ = 1.0. The bacterial inocula were prepared by mixing bacterial cells containing GFP and partitivirus CP constructs in a ratio of 1:2. For the assay with GFP-IR, bacterial suspensions for the GFP, GFP-IR and partitivirus CP constructs were mixed at a ratio of 5:1:5. *N*. *benthamiana* leaves or *Phalaenopsis* sepals were infiltrated with the bacterial inoculum with a needless syringe. GFP intensity in *N*. *benthamiana* was examined at 2 and 5 days post agroinfiltration (dpa) for the assay with and without GFP-IR, respectively. For *Phalaenopsis*, GFP signals were observed using an epifluorescence microscope (Leica DMI 6000B) at 3 dpa. For the assay using onion, the bacterial suspensions were adjusted to OD_600_ = 0.2. The inocula were prepared by mixing of bacterial cells containing GFP and partitivirus CP with or without GFP-IR. The onion bulbs were infiltrated with the bacterial inoculum using a syringe with a needle, and the GFP signals were observed using an epifluorescence microscope at 3 dpa. GFP intensity in *Phalaenopsis* and onion tissues was measured using the Leica Application Suite Advanced Fluorescence (LAS AF) software (Leica Microsystems).

### Preparation of *Rhizoctonia solani* protoplasts

We prepared protoplasts using modified version of Hashiba and Yamada^56^. Three 5-mm-diameter mycelial plugs of *R. solani* subgroup AG4-HGII were transferred to 20-ml V8 broth (1/10 volume of V8 juice, 0.2% CaCO_3_) and grown at 24 °C for 4 days. *R. solani* subgroup AG4-HGII (ATCC 76127) has been originally isolated from sugar beet in the field of Hokkaido, Japan in the laboratory of Plant Pathology, Research Faculty of Agriculture, Hokkaido University, and now maintained in the same lab. Harvested mycelial cultures were homogenized in 60 ml of fresh V8 broth using a Waring blender at 10,000 rpm for 30 s, and aliquots of the homogenized mycelia were incubated at 24 °C overnight in dark. Then, harvested mycelia were washed with osmoticum solution (0.6 M mannitol in McIlvaine buffer, pH 5.14), transferred to osmoticum solution containing 6% (v/v) β-glucuronidase (Sigma-Aldrich), 2% cellulase ‘onozuka’ R-10 (Yakult), 0.5% Macerozyme R-10 (Yakult), and incubated at 32 °C water bath for 3 h with gentle shaking. After passed through the stainless-steel sieve (150 μm pore size), protoplasts were collected by centrifuging at 2000×*g* at 4 °C for 10 min and washed with STC buffer (20% sucrose, 10 mM Tris–HCl, 50 mM CaCl_2_, pH 7.5) two times. The protoplasts were harvested by centrifuging at 2000×*g* at 4 °C for 10 min, then resuspended in STC buffer at a concentration of 10^8^ protoplasts/ml.

### Preparation of nucleic acids for transient expression in fungal protoplasts

The CP gene from each partitivirus was cloned into the CMV-H1 vector^57^, respectively, to be expressed as RNA4A fragments derived from cucumber mosaic virus (CMV). The DNA fragment for RNA4A carrying the partitivirus CP sequence was amplified by PCR using primer pair CM95-4A-5-T7/SSV-12-3, then used as template for in vitro transcription by T7 RNA polymerase (Takara Bio, Shiga, Japan). For inducing RNA silencing against GFP, GFP dsRNA was synthesized as follows: DNA fragments containing partial GFP sequences were PCR-amplified using primer pair EGFP-5-T7-330/EGFP-3-T7-330 with the pMF280-EGFP (GFP-expressing plasmid for fungi) as a template, and the obtained PCR products were transcribed in vitro in both directions using the CUGA 7 in vitro transcription kit (NIPPON GENE CO., LTD, Tokyo). pMF280-EGFP was kindly provided by Dr. Teruo Sone (Hokkaido University, Sapporo, Japan). The plasmid constructs for the RSS activity assay using *Rhizoctonia* protoplasts was shown in Supplementary Fig. S11.

### Transfection of *Rhizoctonia solani* protoplasts

Approximately 10^7^ protoplasts in 100 μl STC were mixed in 20 μl of distilled water containing 5 μg of pMF280-EGFP, 2 μg of CMV RNA4A transcripts and 1 μg of EGFP dsRNA, then held on ice for 20 min. 2 ml of PEG solution (60% PEG #4000, 10 mM Tris–HCl and 50 mM CaCl_2_, pH 7.5) was then added with gentle mixing. The solution was held on ice for another 20 min, then 30 ml of STC was added, and the suspension mixed by inverting several times. The transfected protoplasts were pelleted by centrifugation at 3000×*g* for 10 min, resuspended in regeneration media (1.0 M sucrose, 0.1% yeast extract and 0.1% tryptone) and then incubated at 24 °C for 60 h. GFP fluorescence was observed using an epifluorescence microscope.

### Real-time RT-PCR

Total RNA extracts from leaves of *N. benthamiana* or *Rhizoctonia* protoplasts were first treated with DNase I, and then cDNAs were synthesized using the PrimeScript RT reagent kit (Takara Bio). Real-time RT-PCR was conducted using Powerup SYBR Green master mix (Applied Biosystems). A primer pair S65T-5-168/S65T-3-168 (for *N. benthamiana*) or EGFP-5-152 and EGFP-3-152 (for *Rhizoctonia*) was used to quantify the GFP expression levels. The 60S ribosomal protein L23 gene was amplified using primer pair Nb-L23-5/Nb-L23-3 as the internal control in *N. benthamiana*. The internal transcribed spacer 1 (ITS1) region of rRNA was amplified as an internal control in *Rhizoctonia* protoplasts using primer pair of Rs-rRNA5-100/Rs-rRNA3-100.

### Western blot analysis

Total protein extracts from the infiltration patches on *N. benthamiana* leaves were separated in 12% polyacrylamide gel by SDS-PAGE. The GFP level in each sample was detected by western blot using anti-GFP antibodies. The relative accumulation level of GFP was calculated using Multi Gauge Software (BAS-1000, Fujifilm, Tokyo, Japan).

### Primers

Primer sequences used in this study are shown in Supplementary Table S2.

### Statement for materials

The use of plant materials in this study complies with international, national, and/or institutional guidelines. The seeds of *N*. *benthamiana* were originally obtained with permission from Japan Tobacco Inc. (Tokyo, Japan), which was the former workplace of C. Masuta; the company had distributed tobacco seeds (both wild and cultivated) free of charge to the researchers who use them for non-commercial purposes. Sampling of *Cypripedium macranthos* var. *rebunense* for isolation of OM fungi was conducted with permission of the Ministry of the Environment of Japan.

## Supplementary Information

Supplementary Information.
