# Supplementary material for: Coat protein of partitiviruses isolated from mycorrhizal fungi functions as an RNA silencing suppressor in plants and fungi
Source: Sci Rep. 2022 May 12;12:7855. doi: 10.1038/s41598-022-11403-5 (PMC9098641; doi:10.1038/s41598-022-11403-5)
Supplement: Supplementary file 1 — Supplementary Information. [file 41598_2022_11403_MOESM1_ESM.pdf]

Supplementary Table S1. List of the species used in phylogenetic analysis

Species name	Abbreviation	RdRp	CP
Atkinsonella hypoxylon virus	AhV	AAA61829	AAA61830
Aspergillus ochraceous virus	AoV	ABV30675	ABV30676
Beet cryptic virus 1	BCV1	ACA81389	ACA81390
Beet cryptic virus 2	BCV2	ADP24757	ADP24756
Crimson clover cryptic virus 2	CCCV2	AGJ83769	AGJ83770
Chondrostereum purpureum cryptic virus 1	CpCV1	CAQ53729	CAQ53730
Cryptosporidium parvum virus 1	CSpV1	AAC47805	AAC47806
Dill cryptic virus 2	DCV2	AGJ83771	AGJ83772
Diuris pedunculata cryptic virus	DPCV	AFQ95555	AFY23215
Discula destructiva virus 1	DsV1	AAG59816	AAK13165
Fusarium poae virus 1	FpV1	AAC98734	AAC98725
Fusarium solani virus 1	FsV1	BAA09520	BAA09521
Flammulina velutipes browning virus	FvBV	BAH56481	BAH56482
Gremmeniella abietina RNA virus MS1	GaRV-MS1	AAM12240	AAM12241
Heterobasidion partitivirus 1	HetPV1	ADV15441	ADV15442
Heterobasidion partitivirus 2	HetPV2	ADL66905	ADL66906
Heterobasidion partitivirus 3	HetPV3	ACO37245	ACO37246
Ophiostoma partitivirus 1	OPV1	CAJ31886	CAJ31887
Pepper cryptic virus 1	PePCV1	AEJ07890	AEJ07891
Pepper cryptic virus 2	PePCV2	AEJ07892	AEJ07893
Persimmon cryptic virus	PerCV	CCH50609	CCH50610
Pleurotus ostreatus virus 1	PoV1	AAT07072	AAT06080
Penicillium stoloniferum virus S	PsV-S	AAN86834	AAN86835
Rhizoctonia solani virus 717	RHsV717	AAF22160	AAF40300
Rosellinia necatrix partitivirus 1-W8	RnPV1	BAD98237	BAD98238
Rosellinia necatrix partitivirus 2	RnPV2	BAM78602	BAK53192
Rosellinia necatrix partitivirus 6	RnPV6	BAT24479	BAT24480
White clover cryptic virus 1	WCCV1	AAU14888	AAU14889
White clover cryptic virus 2	WCCV2	AGJ83763	AGJ83764
Ceratobasidium partitivirus Rd4		AOX47570	
Ceratobasidium partitivirus Rd5		AOX47571	
Ceratobasidium partitivirus Rd6		AOX47572	
Ceratobasidium partitivirus Rd7		AOX47573	
Ceratobasidium partitivirus Rd8		AOX47574	
Ceratobasidium partitivirus CPb1			AOX47601
Ceratobasidium partitivirus CPc1			AOX47602
Ceratobasidium partitivirus CPc2			AOX47609
Ceratobasidium partitivirus CPd			AOX47603
Ceratobasidium partitivirus CPe			AOX47604
Tulasnella partitivirus 1		LC649697	LC649698
Tulasnella partitivirus 2		LC649699	LC649700
Tulasnella partitivirus 3		LC649701	LC649702
Human picobirnavirus		BAD98236	
Otarine picobirnavirus		AFJ79071	

Supplementary Table S2. Primers used in this study

Primer name	Sequence (5´-3´)	Purpose
PV1-CP5-Stu	CGAGGCCTATGGCCGATTCCATCACCC	Cloning of CMV-H1-Tulasnella partitivirus 1 CP
PV1-CP3-Spe	CGACTAGTTTACATGGCGACACGAGGG	
PV2-CPn5-St	CGAGGCCTCAAAATGCCTTCCGCTAAGAAACAAC	Cloning of CMV-H1-Tulasnella partitivirus 2 CP
PV2-CPn3-Spe-RV-Sc	CGCGAGCTCGATATCACTAGTTTACTTGTTGAGCACAAATAC	
PV1-CP3-GFP-5	TGGCGACACGAGGGTTG	Cloning of CMV-H1-Tulasnella partitivirus 1 CP-GFP
S65T-3-Xba	GCTCTAGATTATTTGTATAGTTCATCCATGCC	
S65T-5	ATGAGTAAAGGAGAAGAACT	
EGFP-5-T7-330	GCGTAATACGACTCACTATAGGGCATCGACTTCAAGGAGG	Construction of Double-stranded RNA of GFP
EGFP-3-T7-330	GCGTAATACGACTCACTATAGGGTACAGCTCGTCCATGCCGAG	
EGFP-5-152	AGCCGCTACCCCGACCACAT	Real-time RT-PCR for EGFP of *R. solani* protoplast
EGFP-3-152	CGGTTCACCAGGGTGTCGCC	
Rs-rRNA5-100	AGAGTTTGGTTGTAGCTGGCTCCTA	Real-time RT-PCR for reference of *R. solani* protoplast
RS-rRNA3-100	CAAAACATCTGTCTCACAGGT	
CM95-4A-5-T7	ATTAATACGACTCACTATAGGTTTTGTAGTACAGAGTTCAGG	*In vitro* transcription of CMV RNA4A
SSV-12-3	TGGTCTCCTTTGGAAGCCCC	
PV1-CP5-Bam	CGCGGATCCATGGCCGATTCCATCACC	Cloning of pBE2113-Tulasnella partitivirus 1 CP
PV1-CP3-Sc	CGCGAGCTCTTACATGGCGACACGAGGG	
PV2-CPn5-Xb	CGCTCTAGAATGCCTTCCGCTAAGAAACAAC	Cloning of pBE2113-Tulasnella partitivirus 2 CP
PV3-CP5-Xb	GCTCTAGAATGGACGCTCAAAATCCAGT	Cloning of pBE2113-Tulasnella partitivirus 3 CP
PV3-CP3-Sc	CGCGAGCTCCTATTCGTTGGCATCGCG	
S65T-5-168	TCACGGCAGACAAACAAAAG	Real-time RT-PCR for GFP in *N. benthamiana*
S65T-3-168	AAAGGGCAGATTGTGTGGAC	
DP-crp5-1500-St	CGAGGCCTATGGCTCAAGTGACTCCTCC	Cloning of CMV-H1-DPCV CP
DP-crp3-1500-Ml	CGCACGCGTTCAATTGAGACGAGTGTCGG	

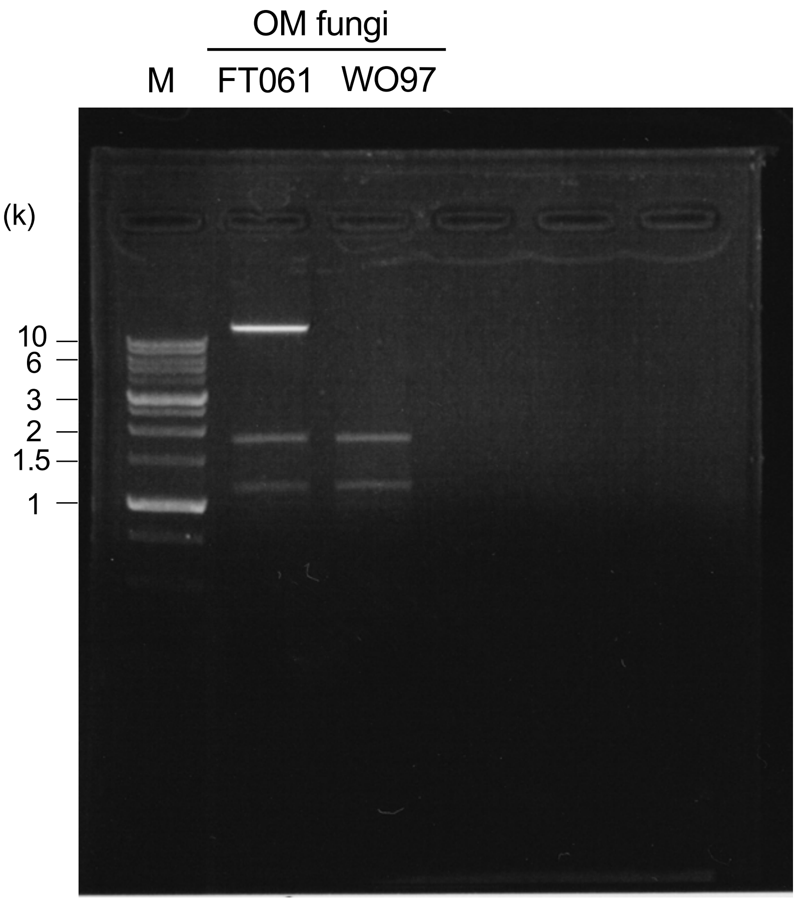

Supplementary Fig. S1. Full-length gel image of Fig.1A. dsRNAs extracted from two OM fungal strains (WO97 and FT061), which were detected in agarose gel electrophoresis.

Tulasnella partitivirus 1

5'UTR

```
RNA1 GCTCTCTGAGACCATCTCA-GTCCTCACCCTAAAGCTTAAACACTCTAGAAGTATTACAC 59
RNA2 GCTCTCTT-GACAACCTCAAGTCCTCACCCTAAAGCTTAAACACTCTAGAAGT-CCA-AC 57
     *******  *** * **** *********************************   * **

RNA1 TCCTCAAACTTGCATCTTAAC-CACAGCCCTGCAA-----TC------- 95
RNA2 TCCTCAAACTCGAATCTTTACTCA-AGCCCTTCAAGTACATCATTAGCA 105
     ********** * ***** ** ** ****** ***     **
```

3'UTR

```
RNA1 ------------------------------------------------------------
RNA2 GCCGCTCCCCCCCCCGCGTCAATAGCTATGTTTTTCACAATCCTCTGTACAATGTATAT 60

RNA1 ---------AGATG-ACG---TGTTTATTAATTTCTTCTT----------------TTCT 31
RNA2 CGTCTGAAGAAATGTGCGCTTTGTTTCTTTCTTTTCTCTTGAGACATTCAACTGTCTTTT 120
              * ***  **   ***** **  ***  ****                ** *

RNA1 TTTATTC----AAATTTGAAAAC-------------- 50
RNA2 TTTATTTTAGAAAATTTTCAAACGCGATTCAACTCGC 157
     ******     ******  ****
```

Tulasnella partitivirus 2

5'UTR

```
RNA1 CGAAAGATCATCTTCGCTGAATTTGAACAGTGCGACAAATCTAAACAATCTACTTTTACT 60
RNA2 CGAAAGATCATCTTCGCTGAATTTGAACAGTGTGCCAAATCTAAACAATCTACTTTTACC 60
     ******************************** * ***********************

RNA1 --------
RNA2 CATTCAAC 68
```

3'UTR

```
RNA1 GGAGTGAGTTTCTTATTGAGCAGGAATTTTTTTCAGTCTCACACCCTTATT----ATGAT 56
RNA2 -----GTGTTCATTGATTAGCCGGAATTTTTTTCG--CTCACTTCCTCGTTTTTGGTGAT 53
          * ***  **  * *** ************   *****  ***  **     ****

RNA1 TTGAGAGCCAATTA--ATCAAATCTTTATTT-ATAATAAGGGT--GTGAGACTGAAA--- 108
RNA2 TGTAGGTTCAAGTCCTACCACCTCCTTATCTCATACCGATCGTTTATGAAAAAAAAAAGG 113
     *  **   *** *   * **  ** **** * ***   *  **   *** *   ***

RNA1 -----
RNA2 ATTTT 118
```

Tulasnella partitivirus 3

5'UTR

```
RNA1  TCCTGAACGAGC-TTCACCAAAGCTTAGATCCCTCAATACAAAGACTTCTCGCTCGCAAT 59
RNA2  -CCTGAACGAGAACTTATCACCCTCAAGCTCCCCAAATACAAAGACTTTTGTCTCGCTCT 59
       **********   * * **      ** ****  ************* *  *****  *

RNA1  CAGAACAGTTTGTACCT------------CGTC-CTACA------------- 85
RNA2  CAGAATAGTTTGTACCTTCCCACCACACCCATCTCTACACACACCAATCGAA 111
      ***** ***********            * ** *****
```

3'UTR

```
RNA1 ------------------------------------------------------------
RNA2 TTCACCACATCTTTCTGGCCACCTTTAGGCCATCTATCAACACTTTCCTCGAGCTCATTT 60

RNA1 --------------------------------------------------------GATT 4
RNA2 GCTCAAACCTCGATCTTGTGTTTTTCTGTTTTCTTTTCCTTTTTCCAGTGAATCATGATT 120
                                                             ****

RNA1 AGAA----------TATTGTTAATTTTTGTTAAAAAAAAAAAA---AA-------TTTTA 44
RNA2 TGAAGTTCTTTCTTTACTTCTCTTTTTCATTCAACACTAACGGTTCAACCCGTTCTTTTA 180
      ***          ** *  *  ****  ** ** *  **      **       *****

RNA1 AAA----------AAAACTCAAC-------------------- 57
RNA2 GGACCCACCATTTGGAATTCAACTTCCAATTTTAACTGAATCA 224
       *            ** *****
```

Supplementary Fig. S2. Alignments of 5' UTR and 3' UTR sequences of RNA1 (RdRp) and RNA2 (CP) of Tulasnella paritivirus 1-3.

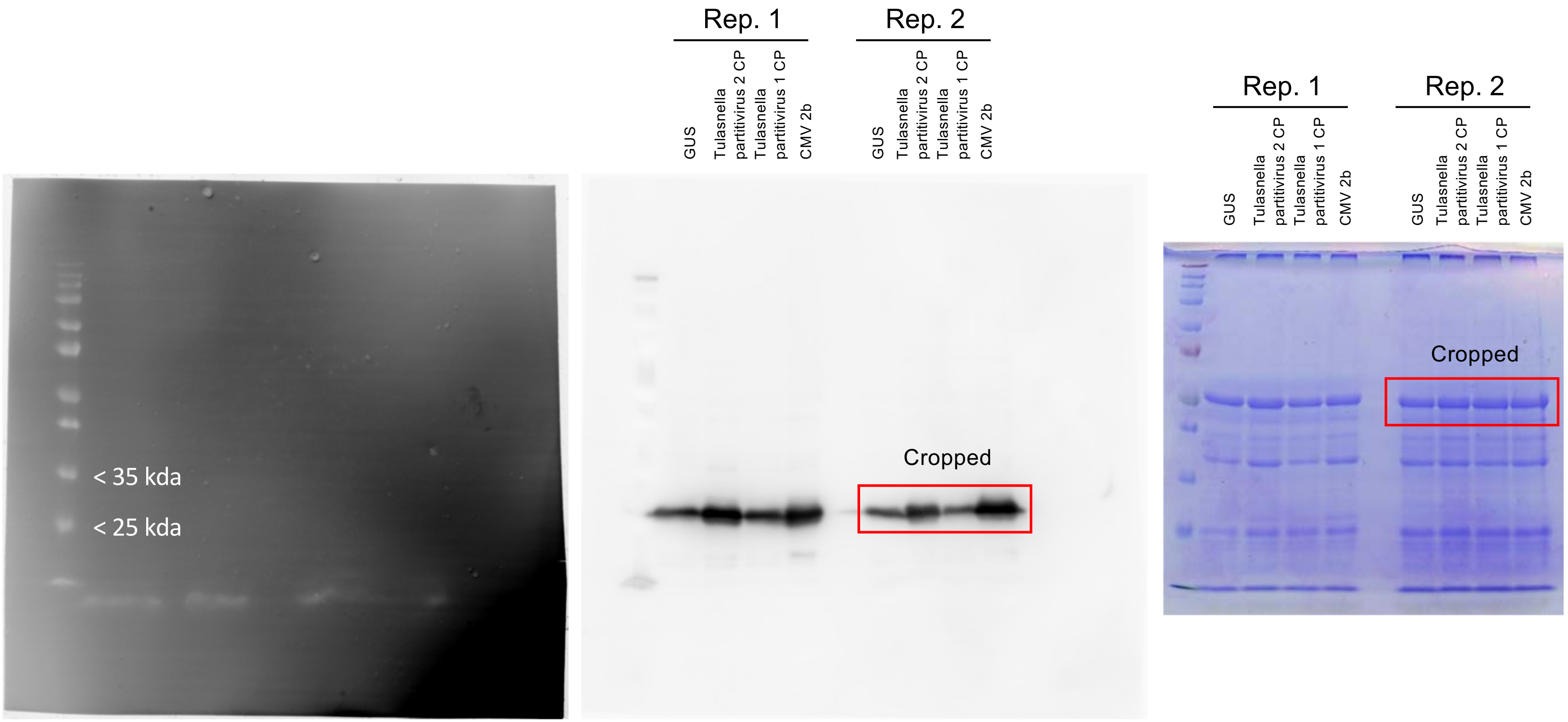

Supplementary Fig. S3. Full-length gel and blot images of Fig. 4C. GFP accumulations in leaves agroinfiltrated with PV1 CP or PV2 CP were analyzed twice (Rep. 1, Rep. 2) by western blotting. Cropped gel/blot image was obtained from Rep. 2 and used in Fig. 4C.
Left, Marker; Middle, western blot, Right, CBB-staining.

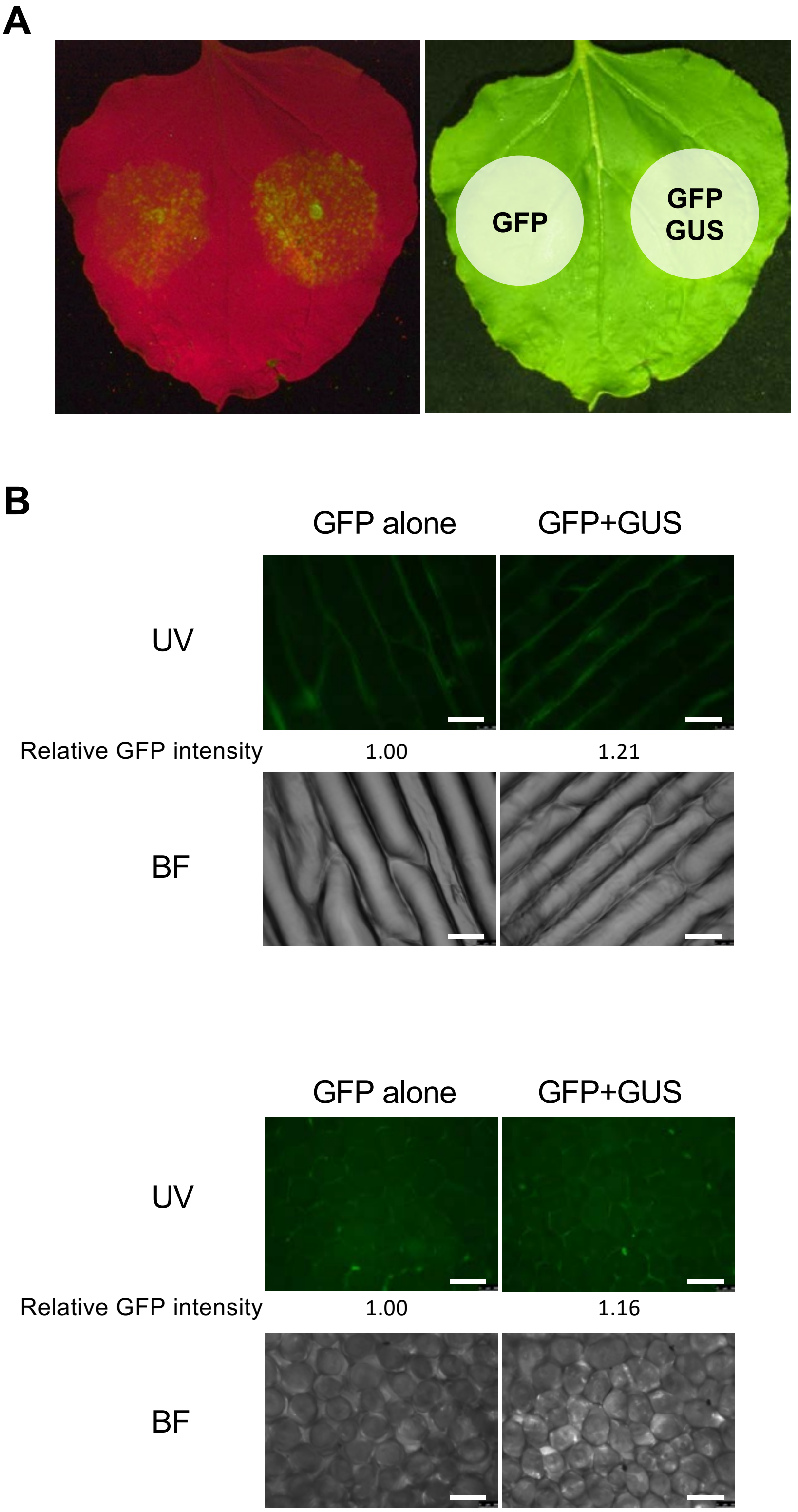

Supplementary Fig. S4. Effect of GUS expression on GFP expression in co-agroinfiltration in plant tissues. For the co-agroinfitration, bacteria containing either the GFP or GUS Ti-plasmids were mixed at a 1:2 ratio. For the control GFP expression, bacteria containing the GFP Ti-plasmid was mixed with bacteria containing no plasmid at a 1:2 ratio. Bacteria were then infiltrated into *Nicotiana benthamiana* leaves (A), onion epidermis (B) and *Phalaenopsis* sepal (C) by agroinfiltration. The GFP expression levels were compared under UV at 5 days post agroinfiltration (dpa) for *N. benthamiana* or at 3 dpa for onion and *Phalaenopsis* tissues using the epifluorescence microscope. The intensity of GFP fluorescence was measured by the LAS AF software (Leica) and expressed as relative values when the control GFP alone was set to 1.0 (B, C). Scale bars: 100 μm.

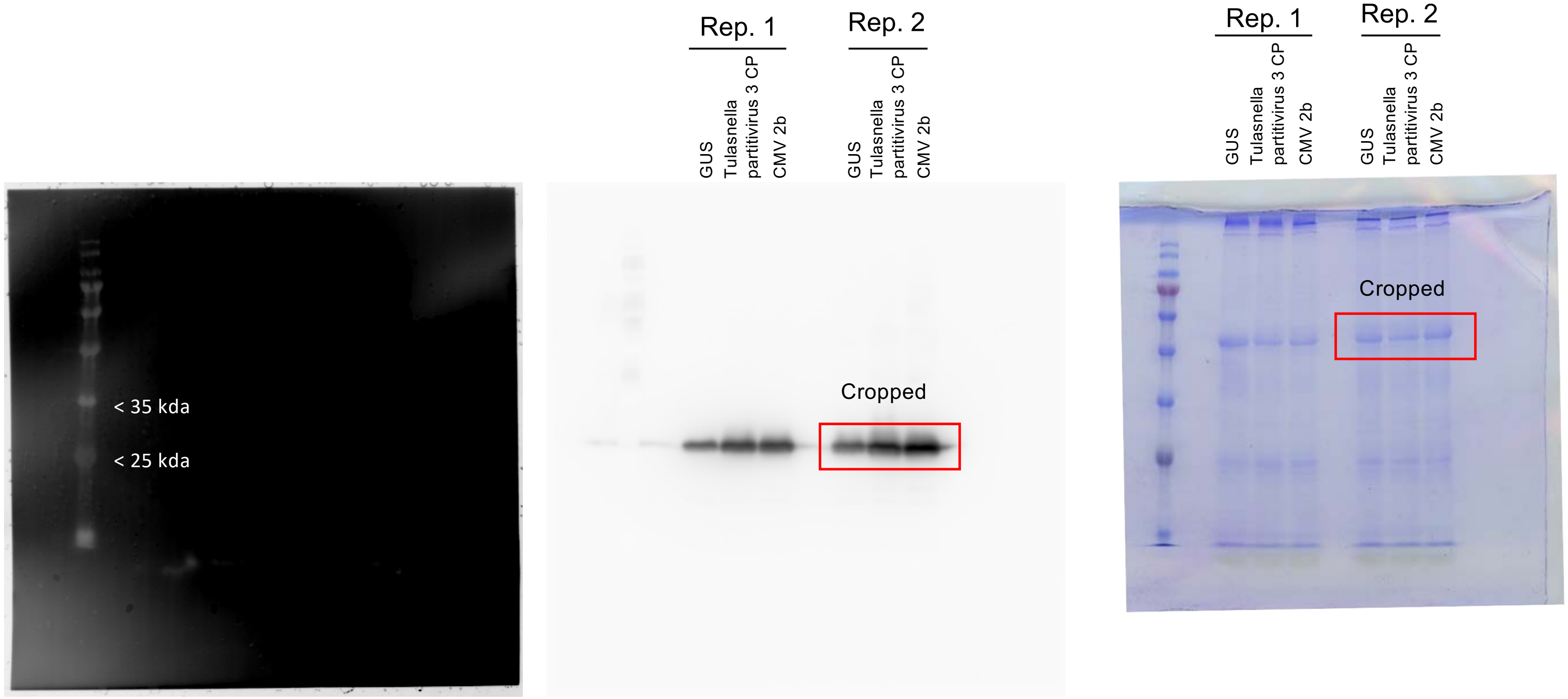

Supplementary Fig. S5. Full-length gel and blot images of Fig. 6A. GFP accumulation in leaves agroinfiltrated with PV3CP was analyzed twice (Rep. 1, Rep. 2) by western blotting. Cropped gel/blot image was obtained from Rep. 2 and used in Fig. 6A.
Left, Marker; Middle, western blot, Right, CBB-staining.

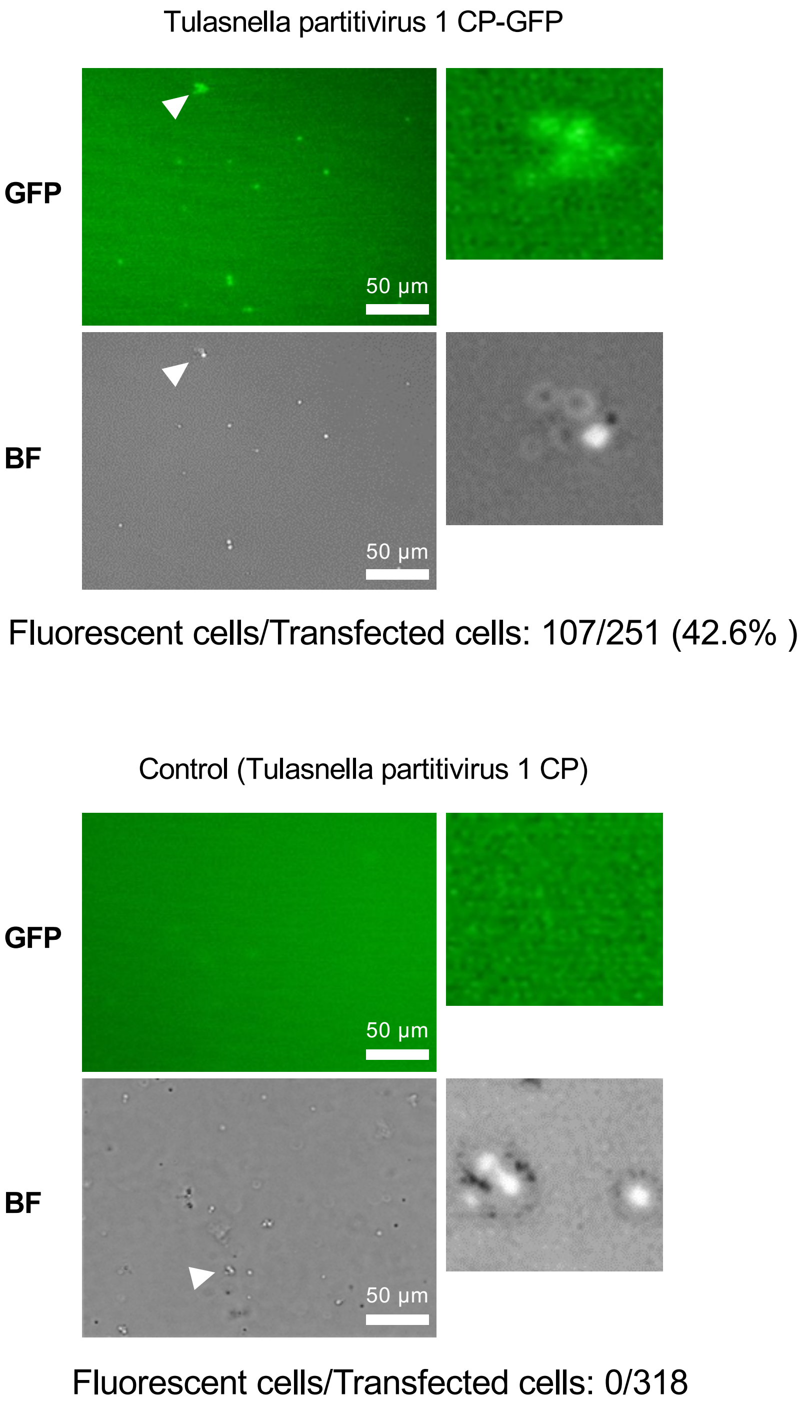

Supplementary Fig. S6. Transient expression of Tulasnella paritivirus 1 CP in *Rhizoctonia solani* protoplasts. Protoplasts of *R. solani* were transformed with RNA transcripts to express Tulasnella paritivirus 1 CP or GFP-fused Tulasnella paritivirus 1 CP. Images were taken using an epifluorescence microscope (Leica DMI 6000B) at 60 h after transfection.

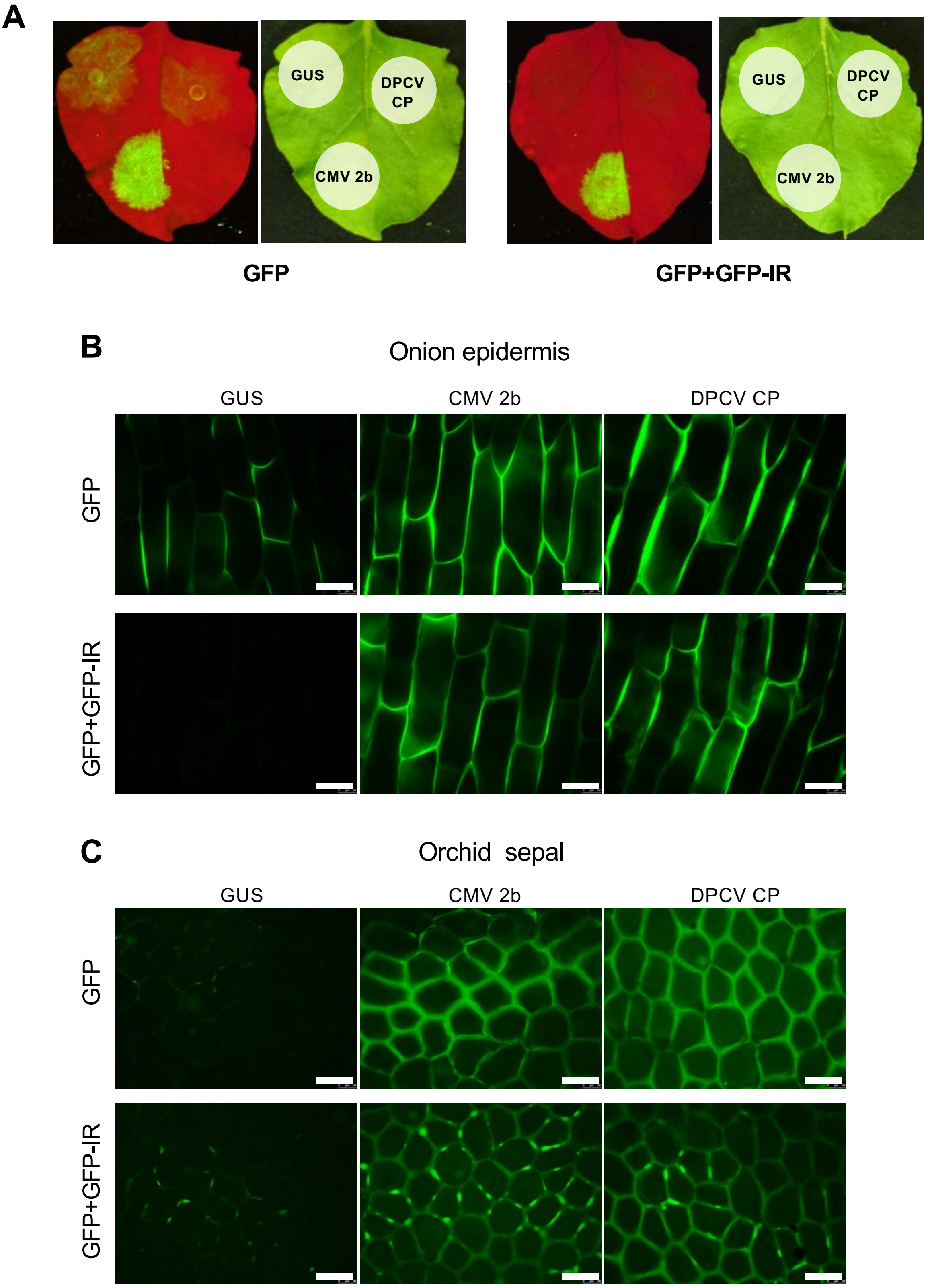

Supplementary Fig. S7. RSS activity of CP of Diuris pendunculata cryptic virus (DPCV) against dsRNA-mediated GFP silencing. RSS activities of DPCV CP expressed with GFP and GFP-IR in *N. benthamiana* leaves (A), onion epidermis (B) and *Phalaenopsis* sepal tissues (C) by agroinfiltration. The GFP expression levels were investigated under UV at 5 dpa for *N. benthamiana*, or at 3 dpa for onion and *Phalaenopsis* using an epifluorescence microscope (Leica DMI 6000B). Scale bars: 50 μm.

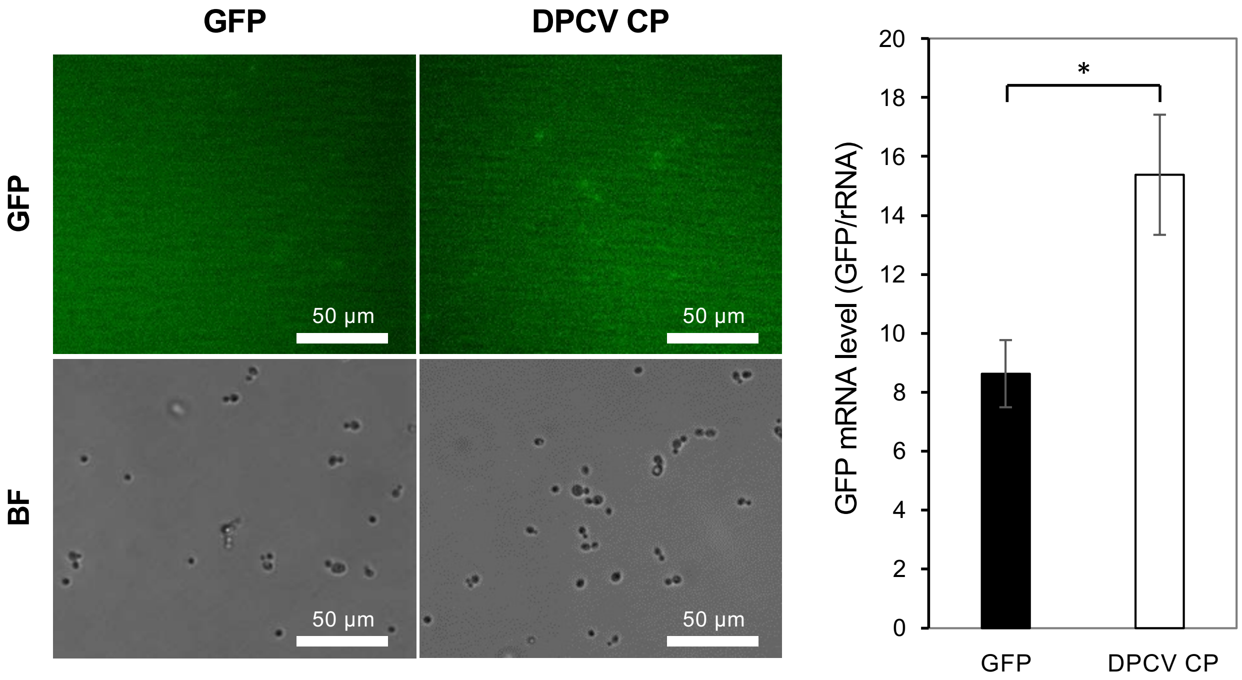

Supplementary Fig. S8. RSS activity of DPCV CP in *Rhizoctonia* protoplasts. (A) graphs of *Rhizoctonia* protoplasts expressing GFP and DPCV CP. Protoplasts of *R. solani* AG4-HG2 were transformed with pMF280, CMV RNA4A harboring DPCV CP and GFP dsRNA. The images were taken at 60 h after transfection using an epifluorescence microscope (Leica DMI 6000B). Right panel: Relative GFP expression levels analyzed by real-time RT-PCR at 60 h incubation. Ribosomal RNA was used as an internal control. Mean values (±SE) were analyzed by Student's *t*-test (*$P < 0.05$).

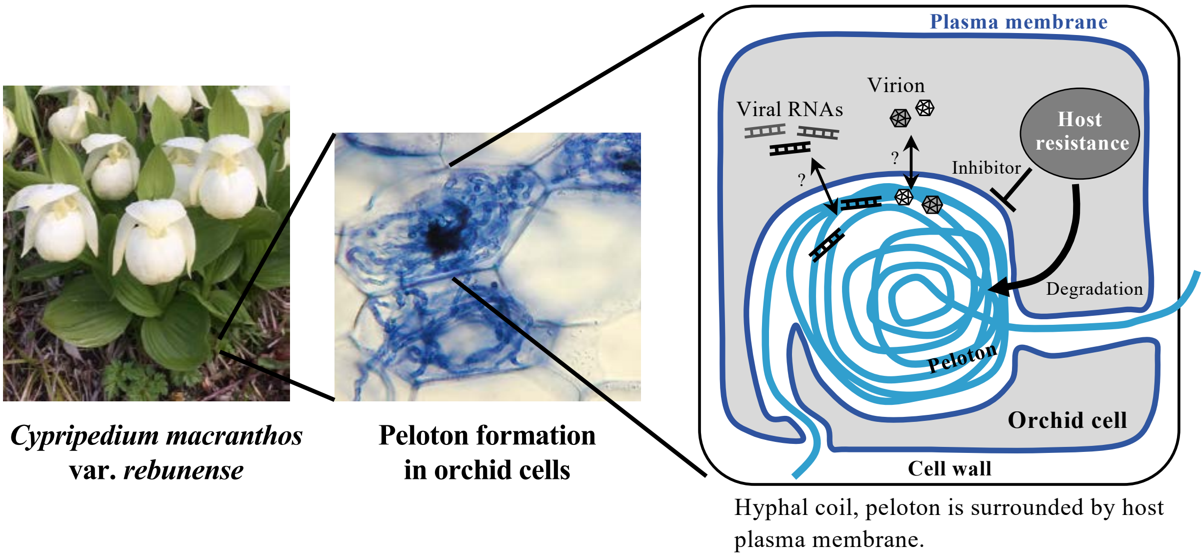

Supplementary Fig. S9. Possible interactions during mycorrhizal formation. The mycorrhizal fungus infects the orchid (e.g., *Cypripedium macranthos* var. *rebunense*) and forms hyphal coils called the peloton inside the orchid cells without breaching the host cell membrane. At the interface between the orchid and fungus, the orchids are expected to deploy resistance responses to control hyphal growth and absorb the nutrients derived from the peloton degradation. Viral particles and RNAs may pass through the cell membranes between the fungus and orchid cells around the peloton.

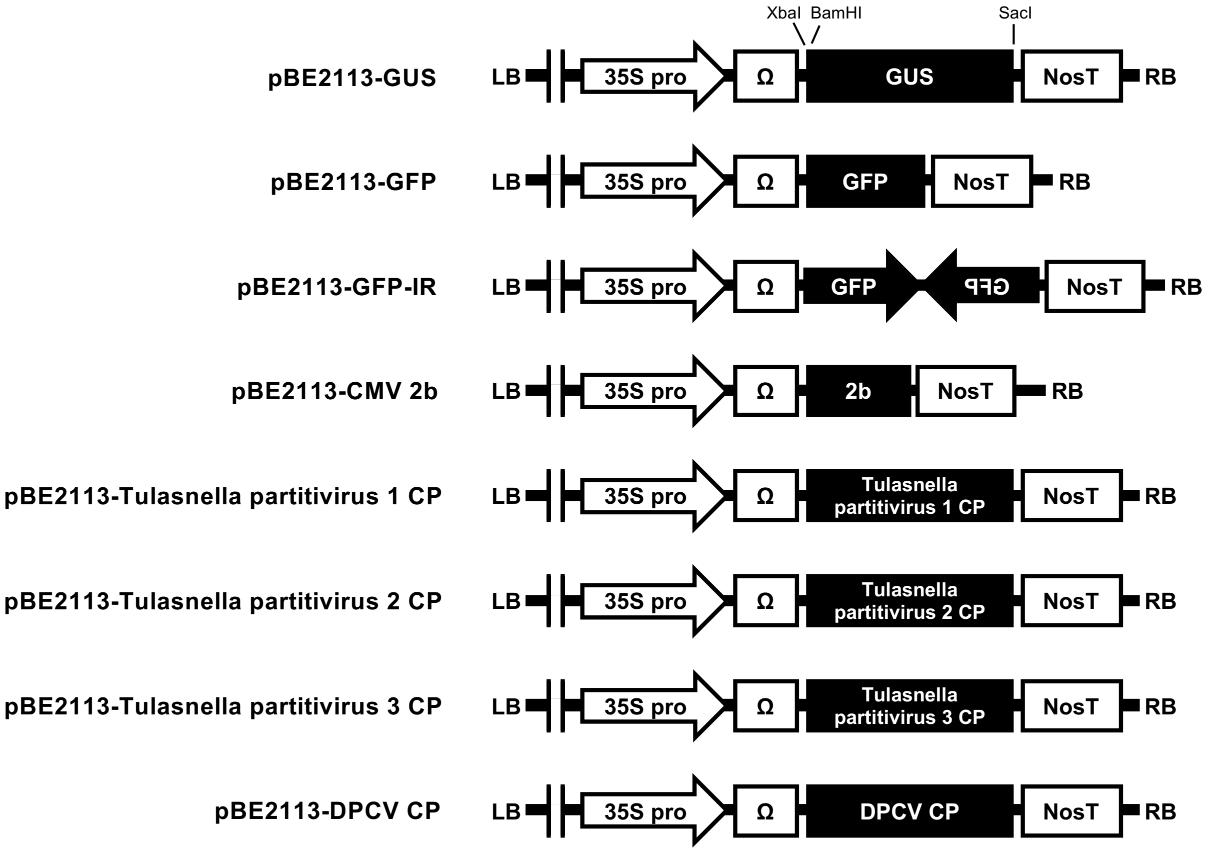

Supplementary Fig. S10. Constructs for RSS activity assay using plant cells. A series of the binary vector constructs expressing GFP or viral proteins were created by replacing the *GUS* gene of pBE2113-GUS Ti-plasmid with virus genes including CMV 2b (2b) and four partitivirus CPs for *Agrobacterium*-mediated transient overexpression. The *GFP* gene and its inverted repeat construct (GFP-IR) were also inserted into the pBE2113 vector for GFP expression and silencing, respectively.

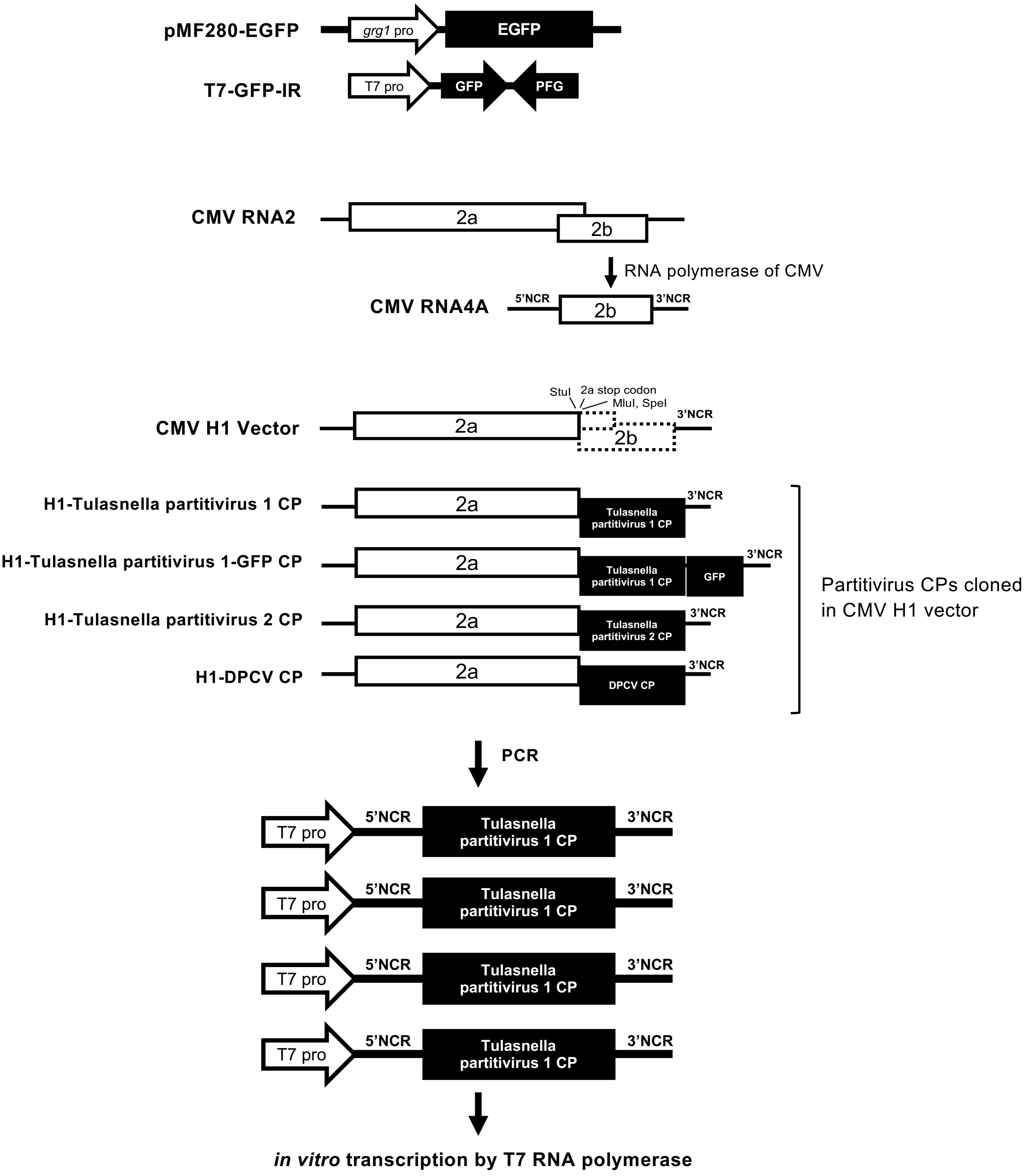

Supplementary Fig. S11. Constructs for RSS activity assay using *Rhizoctonia* protoplasts. (a) For GFP expression in fungal protoplasts, pMF280-EGFP was used in which the *EGFP* gene is expressed under the *Neurospora grg1* gene promoter. GFP-IR RNA was transcribed from the PCR product using T7 RNA polymerase. (b) For the expression of the partitivirus CPs in *Rhizoctonia* protoplasts, each partitivirus CP gene was first inserted into the CMV H1 vector, which was constructed to insert a foreign gene by replacing the CMV *2b* gene. The T7 promoter sequence was attached to the 5' end primer (CM95-4A-5-T7, Supplementary Table S2), which hybridizes upstream of CMV RNA4A. The RNA transcripts, which contain the non-coding region (NCR) sequences of CMV RNA4A, were generated using T7 RNA polymerase and used for the expression of the partitivirus CPs in protoplasts. CMV subgenomic RNAs including RNA4A are efficiently translated both in plants and fungi.
